# How Well Do We Know VPDB—Part 2: Interlaboratory Assessment of Existing *δ* ^13^C_VPDB_ Reference Materials

**DOI:** 10.1002/rcm.10171

**Published:** 2025-12-04

**Authors:** Heiko Moossen, Pharahilda M. Steur, Federica Camin, Bor Krajnc, Anett Enke, Heike Geilmann, Dipayan Paul, Markus Lange, Isabell von Rein, Harro A. J. Meijer

**Affiliations:** ^1^ Max‐Planck‐Institute for Biogeochemistry Jena Germany; ^2^ Centre for Isotope Research, Energy and Sustainability Research Institute Groningen University of Groningen Groningen the Netherlands; ^3^ Terrestrial Environmental Radiochemistry Laboratory, Division of Physical and Chemical Sciences, Department of Nuclear Sciences and Applications International Atomic Energy Agency (IAEA) Vienna Austria; ^4^ Department of Environmental Sciences Jožef Stefan Institute Ljubljana Slovenia

## Abstract

**Rationale:**

In January 2024, the IAEA experts meeting endorsed both *δ* ^13^C scales that are currently used within the scientific community: the Vienna Peedee belemnite (VPDB) *δ* ^13^C scale defined by NBS 19 with a value of +1.95‰ exactly, and the VPDB‐LSVEC scale defined by NBS 19 and the lithium carbonate LSVEC with a value of −46.60‰ exactly. Following the discovery of the instability of the LSVEC material, several expert laboratories independently proposed replacement reference materials (RMs). This study compares these calcium carbonate RMs IAEA‐610, ‐611, ‐612, and USGS44 at the highest level of the metrological traceability chain and recommends values that enable users to realize the VPDB and VPDB‐LSVEC scales.

**Methods:**

The phosphoric acid reaction that is required to evolve CO_2_ from calcium carbonate RMs for isotope analyses is scrutinized by comparing the results of the different apparatuses used in the three participating laboratories. All three laboratories use high‐precision dual‐inlet isotope ratio mass spectrometers and assess the individual instrument offsets in terms of their effects on interlaboratory comparability of samples.

**Results:**

The reported values for IAEA‐610, ‐611, ‐612, and USGS44 on the *δ* ^13^C_VPDB_ scale are −9.114 ± 0.011‰, −30.815 ± 0.011‰, −36.739 ± 0.020‰, and −42.073 ± 0.015‰, respectively. Within their measurement uncertainty they are identical to previously published values. Finally, we provide values on the *δ* ^13^C_VPDB_ and *δ* ^13^C_VPDB‐LSVEC_ scales for some RMs that are routinely used in elemental analysis–isotope ratio mass spectrometry.

## 1 Introduction

Some of the earliest carbon isotope measurements in Earth sciences were made by Alfred Nier in the late 1930s and early 1940s [1, 2]. Since then, the field has grown steadily, and carbon isotope measurements are now commonplace in nearly all scientific fields, from palaeoclimate research [3], to medical [4] and ecological [5], through to atmospheric research [6], to name but a few. Carbon isotope measurements also find applications in food adulteration detection [7], medical forensics [8], and in doping controls [9]. Furthermore, isotopic measurements of, for example, carbon are used to provide information on the atomic weight of carbon within specific substances [10, 11].

Improving standardization and interlaboratory comparability has gone hand in hand with the increased importance of isotope measurements, particularly in fields where such measurements are used to tease out subtle natural variations, or where measurements may come under legal scrutiny. This has led the Forensic Isotope Ratio Mass Spectrometry Network to publish its “Good Practice Guide” [12], and the Global Atmospheric Watch Community, part of the World Meteorological Organization (GAW‐WMO) to formulate minimum requirements for isotope measurements of atmospheric constituents [13].

Unlike fundamental measurement units such as ampere, kelvin, kilogram, and mole, which are now defined through nature constants [14], *δ* ^13^C measurements, as with all stable isotope analyses, are relative abundance ratio measurements, and their values and scales are realized by using specific reference materials (RMs) that are given consensus values. All carbon isotope measurements are expressed using either the Vienna Peedee belemnite scale (*δ* ^13^C_VPDB_) or using the VPDB‐LSVEC (*δ* ^13^C_VPDB‐LSVEC_) scale [15]. These scales have been defined based on the consensus value for one (NBS 19; *δ* ^13^C_VPDB_: +1.95‰) or two (NBS 19 and LSVEC; *δ* ^13^C_VPDB‐LSVEC_: 1.95‰ and −46.6‰, respectively) RMs [16, 17]. Details on the three scales in use for *δ* ^18^O measurements can be found in Camin et al. [15] and Brand et al. [10]. The coexistence of both *δ* ^13^C scales was recently endorsed by the IAEA experts meeting [15]. The International Atomic Energy Agency (IAEA), as the custodian of the stable isotope scales [18], collaborates with expert laboratories to characterize, implement and monitor scale defining RMs. Because calcium carbonates are used as primary and secondary RMs, they are converted to CO_2_ using oversaturated phosphoric acid for high‐precision measurements by gas source mass spectrometry [19, 20].

NBS 19 is quarantined and IAEA‐603 calcium carbonate was introduced as its replacement in 2016 [10, 15, 21]. A second RM for *δ* ^13^C_VPDB_ measurements, the lithium carbonate “LSVEC,” was introduced in 2006, and attributed a consensus value of −46.6‰, to improve interlaboratory measurement comparability [16]. However, recent studies have demonstrated that LSVEC is susceptible to contamination with atmospheric CO_2_, which makes it isotopically unstable [22, 23]. Consequently, its use as an RM for carbon isotope measurements is no longer recommended [24].

In response, expert laboratories have raced to provide an alternative second *δ* ^13^C_VPDB_ scale anchor. Qi et al. [25] introduced the calcium carbonate RM USGS44 and initiated the discussion on the possible need to revise the *δ* ^13^C_VPDB_ scale. Nearly simultaneously, Assonov et al. [26] introduced the three new carbonate RMs IAEA‐610, ‐611, and ‐612. Hélie et al. [27] have highlighted the discontinuity between the *δ* ^13^C_VPDB‐LSVEC_ and the *δ* ^13^C_VPDB_ scales realized by the newly introduced carbonates. Therefore, Assonov et al. [28] have suggested realizing and redefining the VPDB scale using the IAEA RMs IAEA‐603, ‐610, ‐611, and ‐612 and calling it “VPDB2020.” This suggestion has not been adopted by the recent experts meeting [15].

The instability of LSVEC and the introduction of new RMs, together with the discussion on the revision of the *δ* ^13^C_VPDB_ scale underscore the urgent need for additional high precision measurements from expert laboratories. Such efforts are essential to ensure that any future modifications to the *δ* ^13^C_VPDB_ scales are firmly grounded in robust data.

This study reports *δ* ^13^C_VPDB_ values of calcium carbonate RMs USGS44, IAEA‐610, ‐611, and ‐612, providing a unifying set of values that can be used to realize the *δ* ^13^C_VPDB_ scale. The interlaboratory comparability of the phosphoric acid reaction methods used in the three participating laboratories—the Terrestrial Environmental Radiochemistry Laboratory of the International Atomic Energy Agency (IAEA), the Centre for Isotope Research of the University of Groningen (RUG), and the Isotope Laboratory of the Max‐Planck‐Institute for Biogeochemistry (BGC)—is assessed. The scale contraction of the used instruments in the three participating laboratories is also studied using CO_2_ gas RMs. Finally, the results inform the discussion on whether adjusting the carbon isotope *δ* ^13^C_VPDB_ scale is required and to verify the offset between the *δ* ^13^C_VPDB_ and *δ* ^13^C_VPDB‐LSVEC_ scales. This work is part of a larger effort by expert laboratories to improve the isotopic value assignment of RMs and their interlaboratory comparability of measurements. By doing so, it re‐examines the question “How well do we know VPDB” that was posed 16  years ago by Brand et al. [29].

While this study focuses on *δ* ^13^C_VPDB_ data, *δ* ^18^O_VPDB‐CO2_ data are provided and also discussed. However, neither USGS44 nor the IAEA‐610, ‐611, or ‐612 RMs is intended to support or realize the *δ* ^18^O_VPDB‐CO2_ scale [25, 26]. Efforts to provide RMs for, and to verify, the *δ* ^18^O_VPDB‐CO2_ scale are underway, and the results reported here may inform this ongoing effort and future work in the field.

## 2 Methods

### 2.1 Carbonate‐CO_2_ Gas Preparation

All RM CO_2_ syntheses performed in the respective laboratories are listed in Table 1. Subsequently, the CO_2_‐producing phosphoric acid reaction procedures implemented at BGC, RUG, and IAEA are described.

**TABLE 1:** *δ* ^13^C_VPDB_ and *δ* ^18^O_VPDB‐CO2_ average RM values with standard deviations of the mean (all individual data in Data S2). The CO_2_ produced at RUG and BGC was analyzed at BGC only. IAEA produced and analyzed their RM CO_2_ gases independently. *n* indicates the number of individual CO_2_ gases produced per material. The literature values are from Brand et al. [29], Assonov et al. [26], Assonov et al. [21], and Qi et al. [25].

Sample description	CO_2_ producing laboratory	Measurements		*n*	Literature values	
		*δ* ^13^C_VPDB_ [‰]	*δ* ^18^O_VPDB‐CO2_ [‰]		*δ* ^13^C_VPDB_ [‰]	*δ* ^18^O_VPDB‐CO2_ [‰]
NBS 19	BGC	1.95 ± 0.012	−2.20 ± 0.01	10	1.95^a^	−2.2^a^
NBS 19	RUG	1.95 ± 0.011	−2.20 ± 0.05	12		
MAR‐J1	BGC	1.968 ± 0.016	−2.08 ± 0.01	6	1.96 ± 0.01	−2.10 ± 0.01
MAR‐J1	RUG	1.979 ± 0.009	−2.17 ± 0.03	7		
IAEA‐603	BGC	2.461 ± 0.010	−2.40 ± 0.04	19	2.46 ± 0.01	−2.37 ± 0.04
IAEA‐603	RUG	2.472 ± 0.010	−2.51 ± 0.07	9		
IAEA‐603	IAEA	2.460 ± 0.011	−2.36 ± 0.05	66		
IAEA‐610	BGC	−9.125 ± 0.014	−18.91 ± 0.06	6	−9.11 ± 0.01	−18.83 ± 0.04
IAEA‐610	RUG	−9.111 ± 0.001	−19.01 ± 0.01	3		
IAEA‐610	IAEA	−9.108 ± 0.004	−18.80 ± 0.01	4		
IAEA‐611	BGC	−30.824 ± 0.005	−4.22 ± 0.04	7	−30.80 ± 0.01	−4.22 ± 0.05
IAEA‐611	RUG	−30.821 ± 0.010	−4.42 ± 0.03	4		
IAEA‐611	IAEA	−30.807 ± 0.009	−4.22 ± 0.02	6		
IAEA‐612	BGC	−36.755 ± 0.004	−12.04 ± 0.07	6	−36.72 ± 0.02	−12.08 ± 0.06
IAEA‐612	RUG	−36.751 ± 0.006	−12.24 ± 0.08	4		
IAEA‐612	IAEA	−36.724 ± 0.007	−12.07 ± 0.02	6		
USGS44	BGC	−42.077 ± 0.012	−15.74 ± 0.04	12	−42.08 ± 0.01	−15.57 ± 0.07
USGS44	RUG	−42.092 ± 0.009	−15.86 ± 0.03	8		
USGS44	IAEA	−42.064 ± 0.002	−15.63 ± 0.02	6		

^a^ Values are consensus values without uncertainty [15].

#### 2.1.1 Isotope Laboratory of the Max Planck Institute for Biogeochemistry (BGC)

At BGC, CO_2_ gas from CaCO_3_ is prepared offline using the previously described Acid Reaction And Mixing System (ARAMIS; [30]). Briefly, 10 mL of phosphoric acid is placed in the reaction chamber, and 40 mg of calcium carbonate is suspended above the phosphoric acid in the sampling boat using a magnet. ARAMIS is then sealed and evacuated for at least 8 h to at least 1.6 × 10^−7^ mbar removing residual water from all surfaces and from the phosphoric acid. Subsequently, the carbonate is dropped into the phosphoric acid and stirred continuously for 24 h to complete the reaction. Upon completion, the CO_2_ travels through a water trap (ethanol/dry ice) and is frozen into a 300 mL glass flask for a period of 90 min using a liquid nitrogen trap. After this process, a final pressure of at least 2 × 10^−1^ mbar is reached and ~30 mbar of CO_2_ gas is collected.

Each 300 mL glass flask represents one distinct sample with enough gas for at least two separate analyses. Three changes have been made to the system since its implementation. First, the copper reaction chamber and sampling “boat” have been coated with 25 μm of gold to ensure all surfaces in contact with produced CO_2_ and phosphoric acid are as free of water as possible. Second, glass‐sheathed magnetic stirrers are used to stir the reaction. Third, a Peltier element is used to uniformly cool the reaction vessel, providing a stable temperature background against which a heating element maintains the reaction at 25°C ± 0.1°C. Phosphoric acid with a density between 103% and 104% (1.898–1.913 g/cm^2^ at 25°C) is used to avoid adverse isotope effects of acid water content on the produced CO_2_ gas [20].

#### 2.1.2 Isotope Laboratory of the University of Groningen (RUG)

At RUG, CO_2_ from carbonates can be prepared in batches of 10 in McCrea‐type glass reaction flasks [19]. Twenty milligrams of the carbonate sample are placed in each reaction flask, and the side arms are filled with 4 mL of phosphoric acid. The phosphoric acid has a density of at least 1.93 g/mL and is prepared by adding phosphorus pentoxide powder to ortho‐phosphoric acid (85%) until the appropriate density is reached. The flasks are connected to a glass vacuum line and evacuated to 1 × 10^−5^ mbar. Subsequently, the flasks are sealed and disconnected from the vacuum line, and the reaction volume, together with the acid side arm, is submerged into a water bath that is maintained at 25 ± 0.1°C using two Thermo Haake circulators. After 1 h, the reaction is started by tilting the reaction flasks to pour out the phosphoric acid into the reaction chamber containing the carbonate samples. The reaction volumes stay submerged in the water bath overnight. The next day, the flasks are removed from the water bath and connected to the evacuated line, where the produced CO_2_ passes over a water trap and is then trapped in a sample flask using liquid N_2_. Once the pressure is in the range of 1 × 10^−1^ mbar, a vacuum pump is used to gently extract the rest of the CO_2_ from the phosphoric acid until the pressure stabilizes at 1 × 10^−3^ mbar. Subsequently, the sample vial is isolated from the pump and flame‐sealed. The amount of gas produced from each flask is enough for multiple analyses.

#### 2.1.3 Isotope Laboratory of the International Atomic Energy Agency (IAEA)

At the IAEA laboratory, CO_2_ gas from carbonates is prepared offline using a MULTIPREP 2000 gas preparation system (Scientific Solutions Ltd., New Zealand) which is capable of producing 20 individual gas samples per batch. It consists of 20 pairs of reaction vessels (flasks with side arms) and their corresponding 20 expansion vessels (glass containers with a pneumatic valve). Each reaction vessel is connected to a water trap through which the sample from the reaction vessel is expanded to the expansion vessel. Ten milligrams of calcium carbonate sample are placed in the reaction vessel and 2 mL of 103%–104% phosphoric acid in the side arms. Reaction flasks are held at a controlled temperature of 25°C and evacuated to high vacuum overnight. The reaction is started by tilting the flasks in order to pour the phosphoric acid from the side arms over the carbonate samples, and the mixture is shaken for 24 h. After the reaction is complete, the produced CO_2_ is expanded from the reaction vessel through the water trap into the expansion vessels. Expansion vessels are then manually connected to the multi‐port inlet system on the DI‐IRMS. Each vessel represents one distinct sample with enough gas for one bellows filling and for measurement of at least three repetitions.

### 2.2 Isotopic Analysis

In this study, all isotope results derived from carbonate phosphoric acid reactions are reported on the *δ* ^13^C_VPDB_ and *δ* ^18^O_VPDB‐CO2_ scales. The *δ* ^18^O_VPDB‐CO2_ scale is a virtual scale realized by normalizing all sample values to CO_2_ evolved from NBS 19 carbonate with oversaturated phosphoric acid at 25°C [17, 31, 32]. Because *δ* ^13^C_VPDB_ and *δ* ^13^C_VPDB‐CO2_ values are identical, we report carbonate *δ* ^13^C results on the *δ* ^13^C_VPDB_ scale. All elemental analysis–isotope ratio mass spectrometry (EA‐IRMS) measurements are also reported on the *δ* ^13^C_VPDB_ scale. Delta values are calculated using Equation (1):

(1) $$\delta^{i}E_{\text{Sample}\left(\text{VPDB}\right)}=\left(R_{\text{Sample}}/R_{\text{Reference material}}\right)-1$$

where *δ* ^i^E stands for the delta ^13^C or ^18^O value and *R* denotes the ^13^C/^12^C or ^18^O/^16^O ratio of the sample and RM [33].

The IUPAC approved expression is *δ*(^13^C) and an alternative expression is *δ*(^13^C/^12^C) [15, 34]. The *δ* values are usually small numbers and are therefore multiplied by a factor of 1000 and reported in permille (symbol ‰).

#### 2.2.1 BGC—Isotope Ratio Measurements

BGC employs a dual‐inlet stable isotope ratio mass spectrometer (DI‐IRMS; MAT253, Thermo Fisher Scientific, Bremen, Germany) for carbon and oxygen stable isotope analyses of CO_2_. Following Verkouteren et al. [35], the ion source settings and method were optimized to reduce the sample/reference gas cross‐contamination [36]. The ion source emission is set to 0.8 mA and sample/reference gas (also known as “monitoring gas” that acts as a mediator between daily sample analyses [37]) signal intensities are limited to 3000 mV. The idle time between switching the change‐over valve (COV) is 15 s. Sample, working standard and quality control CO_2_ gases are filled into the left bellows and analyzed against the reference gas (filled into the right bellows) following the principle of identical treatment [37]. A daily sequence consists of two working standard measurements, one at the start and end of the sequence, three samples, and one quality control measurement. The SSH [38] algorithm is employed by the IsoDat 3.0 software (Thermo Fisher Scientific) to perform the online ^17^O correction. If IsoDat 3.0 is used for the ^17^O correction, it is important to check that the IUPAC‐recommended parameters by Brand et al. [31] (λ: 0.528; k: 0.01022461) are implemented. This can be done in the Registry Editor. Delta values provided by IsoDat are corrected offline for daily reference gas drift (linear model drift correction) and cross‐contamination (eta (*η*)‐correction; Data S1), which occurs at the COV between the reference and sample gas.

The COV switches 20 times between the sample and reference gas, resulting in a standard deviation (S.D.) for each of three measurements of a bellows filling. The propagated standard uncertainty of the three measurements of each bellows is calculated by propagating the uncertainty of individual measurements with the S.D. of the *η*‐correction and the S.D. of the normalization to the *δ* ^13^C_VPDB_ scale. In the next step, an average value of the three measurements of a bellows with its propagated standard uncertainty is calculated, thus providing a measurement value and uncertainty for each individual measurement (Data S2). The uncertainty propagation employed at BGC is described in Data S1 and follows the “Evaluation of measurement data ‐ Guide to the expression of uncertainty in measurement” guidelines (GUM; [39]).

Using ARAMIS, 10 NBS 19‐CO_2_ gas samples were created in early 2022. These NBS 19‐CO_2_ gases were analyzed in 10 sequences against 20 working standard CO_2_ gases “Cecily 2019a” over a month‐long period. The *δ* ^13^C_VPDB_ and *δ* ^18^O_VPDB‐CO2_ values of “Cecily2019a” are −4.045 ± 0.005‰ and −13.745 ± 0.007‰, respectively, scaled to 10 NBS 19 CO_2_ syntheses. Individual unscaled measurement values of NBS 19 and Cecily2019a are provided in Data S2.

Each daily measurement sequence includes a quality control measurement of the CO_2_ gas “Simon” with *δ* ^13^C_VPDB_ and *δ* ^18^O_VPDB‐CO2_ isotope values of −37.998 ± 0.006‰ and −35.804 ± 0.010‰ (September 2021–February 2025, respectively, Data S1). The large isotopic difference between the working standard and quality control gas ensures that even small instrumental analysis problems can be identified. The standard deviations of the *δ* ^13^C_VPDB_ and *δ* ^18^O_VPDB‐CO2_ quality control measurements between September 2021 and February 2025 are a factor of three smaller than the average standard uncertainty of individual quality control measurements. One reason for this is that the average standard uncertainty of individual measurements includes the uncertainty introduced by the *η*‐correction and the normalization to the VPDB scales, while the standard deviation includes the variability of measurements around the mean only. This indicates that the approach of estimating the uncertainty of a measurement of a single bellows filling at the BGC‐IsoLab is conservative.

To link the calcium carbonate RM values reported here to the suite of organic RMs that are used for carbon isotope measurements, and to verify the scale differences between the VPDB and VPDB‐LSVEC scales, EA‐IRMS was employed following the method and techniques described previously [37, 40]. BGC uses a DeltaPlus IRMS (Thermo Fisher Scientific) coupled to a 1100 ce EA analyzer (Carlo Erba, Rodano, Italy) via a ConFlo III Open‐Split interface (Thermo Fisher Scientific). Measurement sequences and post‐measurement blank, linearity, and drift corrections were performed according to Werner and Brand [37]. The *δ* ^13^C_VPDB_ measurements were normalized using a 2‐point calibration equation with NBS 19 (+1.95‰) and USGS44 (−42.073 ± 0.015). This is the USGS44 value and uncertainty determined in this study (see Section 3).

#### 2.2.2 RUG—Isotope Ratio Measurements

At RUG, a PrecisION DI‐IRMS (Elementar Analysensysteme GmbH) was used for the analysis of the *δ* ^13^C_VPDB_ and *δ* ^18^O_VPDB‐CO2_ composition in CO_2_ samples. The ion source is operated at around 3.8 kV and tuned such that the major beam, *m/z* 44, is measured at 25 nA. Every measurement comprised 6 reference‐sample changeover pairs with a 12 s wait time before integrating the reference/sample data for 25 s. The instrument is controlled, and the data are analyzed using the LyticOS software suite, provided with the instrument. Following every sample measurement, a background scan for water and argon contamination was performed to ensure both levels matched the background values corresponding to the machine reference. Only for measurements reported in this work, every sample introduced into the IRMS was measured twice.

Every measurement batch comprised a local CO_2_ reference that was measured five times throughout the batch to account for any instrumental drifts. The measurement repeatability over one batch is typically 0.01‰ and 0.03‰ for *δ* ^13^C and *δ* ^18^O, respectively. Samples are calibrated by a multipoint calibration. The isotopic composition of the sample was calculated using the iterative ^17^O correction approach recommended by IUPAC [31]. This approach is identical to the one described for BGC measurements. During this measurement exercise, when CO_2_ samples produced from carbonate RMs at Jena were measured, the same RMs were also freshly prepared in triplicates and measured in the same batch. All samples are normalized against 12 NBS 19 CO_2_ gases produced at RUG (Data S2). RUG uses two local CO_2_ laboratory standard gases with *δ* ^13^C_VPDB_ and *δ* ^18^O_VPDB‐CO2_ values of −3.3 ± 0.03‰ and −13.5 ± 0.03‰, and −30.4‰ ± 0.02 and −31.5 ± 0.03‰, respectively. These laboratory gas standards are only used for stability checks and to monitor possible drift of the IRMS, not for calibration.

#### 2.2.3 IAEA—Isotope Ratio Measurements

For a detailed description of the analytical and data handling procedure implemented at the IAEA, the reader is referred to Assonov et al. [21]. In short, the IAEA uses a MAT253 DI‐IRMS (Thermo Fisher Scientific, Bremen, Germany) with the following measurement settings to reduce the cross‐contamination effect. The high voltage is set to 6 kV; the ion source emission is set to 0.7 mA; the VISC valve is open; the sample/reference gas signal intensities are set to 4500 mV; and the idle time is set to 16 s. The raw *δ* ^45^ *R* and *δ* ^46^ *R* values (provided by the IsoDat software) were scaled to VPDB via 1‐point calibration relative to six NBS 19‐CO_2_ syntheses. Values rescaled to primary RMs were corrected for cross‐contamination according to Srivastava and Verkouteren [41] and then used for *δ* ^13^C and *δ* ^18^O calculations using the iterative ^17^O correction approach with parameters recommended by IUPAC [31]. Internal quality control samples were used to demonstrate the absence of day‐to‐day drifts during the measurement period. The assessment of combined uncertainty included: repeatability of measurements of samples and the measured RM, assigned uncertainty of the RM, and uncertainty introduced by the *η*‐correction. All uncertainty components were taken as 1 standard deviation and combined in squared form following JCGM [39].

### 2.3 Statistical Analyses

The two one‐sided test (TOST) equivalence test is used to compare the data from the three laboratories [42, 43, 44]. The TOST procedure evaluates two hypotheses. One is whether the spread between the means of two sets of measurements is small enough that the populations could be considered equivalent within accepted criteria. This is particularly useful where measurements from two laboratories may indeed be statistically different from each other owing to high measurement precision, for example, but where that difference has no practical or scientific relevance for real‐world applications [43]. To test the equivalence hypothesis, upper and lower equivalence bounds are defined. If the mean difference between the two groups, along with its 90% confidence interval (CI), falls entirely within these bounds, the two groups are deemed equivalent within the specified limits (Figures 2, 3, and 5). This outcome indicates that the datasets generated by the two laboratories for the same material are close enough to meet external accuracy requirements. In this study, the *network compatibility goals* of ±0.01‰ for *δ* ^13^C‐CO_2_ and ±0.05‰ for *δ* ^18^O‐CO_2_ measurements as defined by the GAW‐WMO community (table 1 in [13]), are used to delineate external accuracy requirements. These very strict boundaries represent, to the authors' knowledge, the highest accuracy requirement in any field of isotope research. While the datasets from two laboratories may be equivalent within set boundaries, this does not necessarily mean that they can be considered identical [43]. The second hypothesis tests whether the CIs of two datasets overlap, i.e., whether the data produced in one laboratory overlaps with the data from the other and, in doing so, are identical. This is the case when the 90% CI of the mean of both datasets intersects zero in the TOST procedure (Figures 2, 3, and 5). Note that the CI of the TOST statistic increases when the data are noisy or only a few measurements have been made [43].

The IAEA‐603, ‐610, ‐611, 612, and USGS44 datasets from the three laboratories are unified, and average values are reported (Table 3). To do this, the values associated with the phosphoric acid reactions from all laboratories are taken into account even though they do contribute to interlaboratory variability of the data. However, the average values only consider measurements done at BGC and IAEA as the RUG instrument in its present clumped isotope configuration is not suitable for the 1‐point calibration due to its insufficient abundance sensitivity (see discussion below). The linear pool model of the NIST consensus builder software (https://consensus.nist.gov/app/nicob) was used to calculate average consensus values from individual measurements done at BGC and IAEA (Data S2), based on the mean values and their standard deviations from each laboratory (Table 1). The linear pool model assigns Student's *t* distributions to the datasets according to their degrees of freedom and thereby weighs the contributions of individual datasets to the final mean value based on the amounts of measurements made in each laboratory (for further details, the reader is referred to the User Manual of the NIST consensus builder, https://consensus.nist.gov/app/nicob).

## 3 Results and Discussion

### 3.1 Cross‐Contamination Correction

The primary RM that defines the *δ* ^13^C_VPDB_ scale is NBS 19 calcium carbonate. Even though NBS 19 is quarantined some expert laboratories, including the participants of this study, retain NBS 19 for special calibration purposes. The secondary RMs IAEA‐603 and USGS44 are calibrated against NBS 19 [21, 25], and IAEA‐610, ‐611, and ‐612 are normalized against IAEA‐603 [26]. Although these secondary RMs are available, the independent assessment of their *δ* ^13^C_VPDB_ values, including the interlaboratory comparability of the measurements, requires that only the primary RM NBS 19 is used as a calibrant. When using NBS 19 derived CO_2_ gas as a single VPDB scale anchor, the instrument‐specific scale contraction caused by sample gas/reference gas cross‐contamination (*η*‐effect) needs to be quantified [36].

Sample gas/reference gas cross‐contamination is caused by adsorption of gas molecules on the ion source surfaces, ion implantation and sputtering [35, 36], and mixing of sample and reference gas at low idle times. A low abundance sensitivity can also lead to scale contraction as reported by RUG in this study (see discussion below). The ion source surfaces that play the largest role are the focusing elements that define the shape of the ion beam as it enters the sector field [45]. During operation, these focus lenses, particularly the exit slit (α‐slit), are impacted by sample ions and molecules and are subject to change throughout their lifetime. Operating the ion source at low electron emission (0.8 mA at BGC) reduces cross‐contamination and wear on the ion source lens surfaces.

**FIGURE 1:**
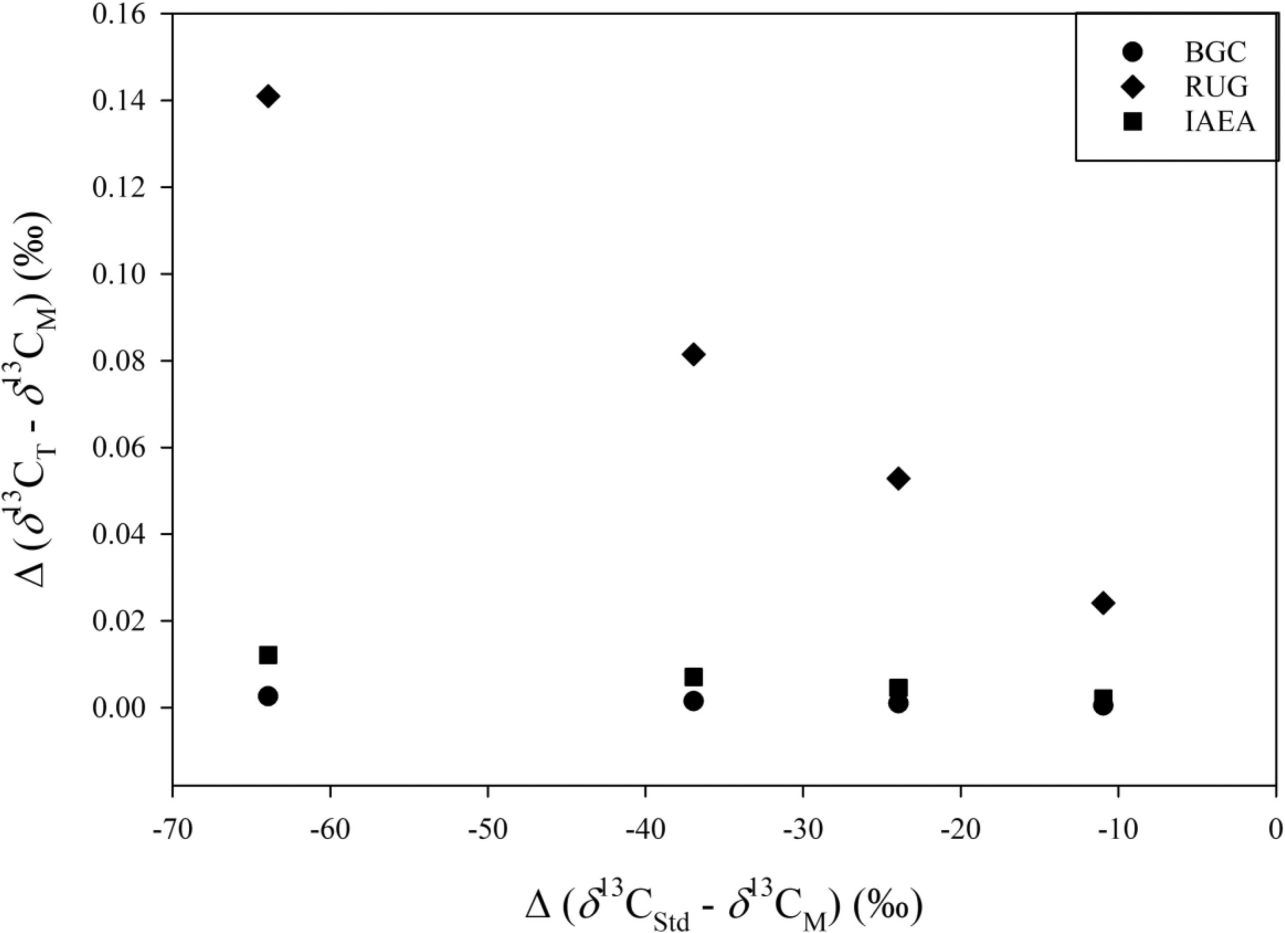
Modelled influence of the eta correction (offset between the true, eta corrected value (*δ* ^13^C_T_) and the measured value (*δ* ^13^C_M_)) on *δ* ^13^C measurements of samples relative to their isotopic distance from the primary RM (NBS 19, *δ* ^13^C_VPDB_: 1.95‰) using the reported *δ* ^13^C_*η*_ values from the three laboratories in this study. The isotope values chosen for *δ* ^13^C_M_ in this visualization are −9‰ (representing atmospheric carbon dioxide, [46]), −22‰ (representing C3 plant metabolic processes, [11]), −35‰ (representing C3 plant leaf wax components, [47]), and −62‰ (representing biogenic sources to atmospheric methane, [48]).

Following Verkouteren et al. [35], three idle time experiments were conducted at BGC, and the average calculated *η*‐factors for a COV idle time of 15 s (*δ* ^13^C_*η*_: 0.00004 ± 0.00012; *δ* ^18^O_*η*_: 0.00035 ± 0.0007) were used to correct sample measurements in this study (Figure 1, Data S1). The uncertainty in the *η*‐factors is the largest contributor to the uncertainty budget and covers the possibility of a negative *η*‐factor due to the experimental setup of the idle time experiments (Data S1). In the case of USGS44 (*δ* ^13^C_VPDB_: −42.07 ± 0.01, this study), the cross‐contamination correction introduces an uncertainty of ±0.0075 to the measurement of a single bellows filling. The 15 s idle time is used as longer idle times have an adverse impact on sample throughput during routine operation. At RUG, cross‐contamination in all measurements was corrected using the introduction of isotopically enriched CO_2_ into the sample below [36]. This gave values of *δ* ^13^C_*η*_ = 0.0022 and *δ* ^18^O_*η*_ = 0.0054. In spite of this correction, scale contraction was still observed. This was further investigated by studying a high‐resolution mass scan. On the RUG instrument (PrecisION), while using the Faraday cup array for clumped isotopes (cups for masses 44–49), significant signal tailing from one cup into the next, i.e., low abundance sensitivity [49] was observed. The structure of this tailing is not smooth enough to produce a reliable correction when using a 1‐point calibration to value assign RMs that are isotopically far away from the primary RM. Therefore, only RUG measurements of NBS 19 and IAEA‐603 are reported here. The isotope values are close enough together so that the *η*‐correction is negligible (Figure 1). These results can therefore be used to study the phosphoric acid reaction. At the IAEA, four idle time experiments were conducted following the Verkouteren et al. [35] approach. Each experiment consisted of several measurements with 16, 150, and 300 idle times. Because routine measurements at IAEA are done with an idle time of 16 s, average calculated *η*‐factors of four experiments for a COV idle time of 16 s were calculated. Values of 0.00019 ± 0.00010 and of 0.00040 ± 0.00009 were obtained for *δ* ^13^C_*η*_ and *δ* ^18^O_*η*_, respectively, and used for the cross‐contamination correction of the results.

Figure 1 shows that the cross‐contamination effect can vary drastically among instruments and that it increases with the isotopic distance between sample and standard (*δ* ^13^C_Std_–*δ* ^13^C_M_). This is an important consideration when analyzing very ^13^C‐depleted samples such as biogenic methane (global mean −62‰, [48]). Not correcting the scale contraction would cause a 0.01‰ offset in the values produced by the two identical IRMS instruments used in this study at IAEA and BGC. This is not inconsequential, particularly when considering the WMO‐GAW network compatibility goal of 0.02‰ for measurements of carbon isotopes of atmospheric methane [13]. An easy way to avoid having to correct for scale contraction is to make sure that the isotope values of the RM and samples are as identical as possible. Even a relatively large cross‐contamination as observed for the RUG instrument only causes an offset of 0.001‰ when analyzing the *δ* ^13^C_VPDB_ value of IAEA‐603 (*δ* ^13^C_VPDB_: +2.46‰) relative to NBS 19.

The *η*‐factor values of the BGC instrument reported in this study are in good agreement with the average *η*‐factor values that were reported for the same instrument in 2018 and 2019 (*δ* ^13^C_*η*_: 0.00046 ± 0.0005; *δ* ^18^O_*η*_: 0.00097 ± 0.001 and *δ* ^13^C_*η*_: 0.00032 ± 0.0005; *δ* ^18^O_*η*_: 0.00078 ± 0.001; [25]). Because the ion source settings are chosen to reduce cross‐contamination as much as possible, this result is not surprising. However, it is still recommended that the *η*‐factors be determined when high precision measurements are made over a large range of isotopic values without the use of a second scale anchor.

### 3.2 Interlaboratory Comparison of RM Isotope Results

Numerous laboratories were involved in providing the best measurements for the NBS 19 when it was introduced [50]. Most of these laboratories used diverse local standard calcium carbonates as RMs and the standard deviation of the average value of NBS 19 from all laboratories was close to 0.1‰ (after removal of outliers) for *δ* ^13^C and *δ* ^18^O measurements. Since then, further research has shown that interlaboratory comparisons of stable isotopes of CO_2_ remain challenging [25, 29, 51]. Assuming that different laboratories use the same primary RM, challenges still arise from the preparation of CO_2_, i.e., the phosphoric acid reaction and instrumental discrepancies such as the above‐discussed instrument‐specific scale contraction. In the following section, we compare the isotope results of the calcium carbonate RMs IAEA‐603, ‐610, ‐611, and ‐612 and MAR‐J1, an in‐house RM used by BGC to realize the JRAS‐06 scale [52]. Table 1 shows the results from measurements conducted at BGC and IAEA and from phosphoric acid reactions performed at all three laboratories: BGC, RUG, and IAEA.

#### 3.2.1 BGC–RUG Comparison

**FIGURE 2:**
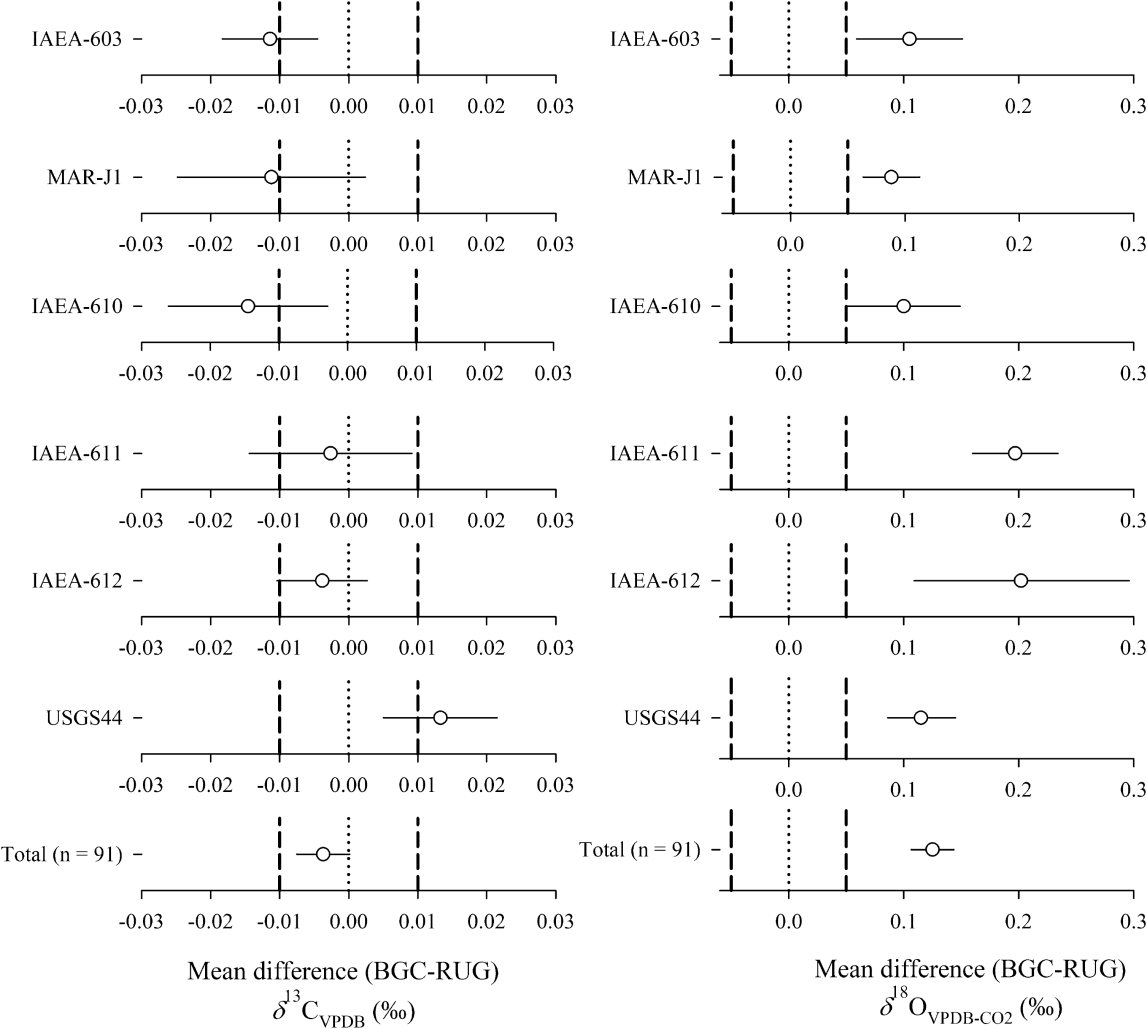
Assessing the equivalence of the stable isotope signatures from carbonate‐CO_2_ produced at BGC and RUG and measured at BGC based on two one‐sided tests (TOSTs). The data points indicate the mean difference between the datasets with associated 90% confidence intervals. The vertical dashed lines indicate the lower and upper boundaries as defined by the WMO‐GAW network compatibility goals (*δ* ^13^C_VPDB_: ±0.01‰; *δ* ^18^O_VPDB‐CO2_: 0.05‰). For the bottom two graphs, all datasets are normalized to zero by subtracting each individual measurement from the mean of all measurements from both laboratories for each RM. If the confidence intervals cross the boundaries, then the data is not equivalent within those boundaries. The zero point is indicated by the dotted line. If the confidence interval crosses the zero point, then the respective datasets from the two laboratories are not significantly different from each other.

**FIGURE 3:**
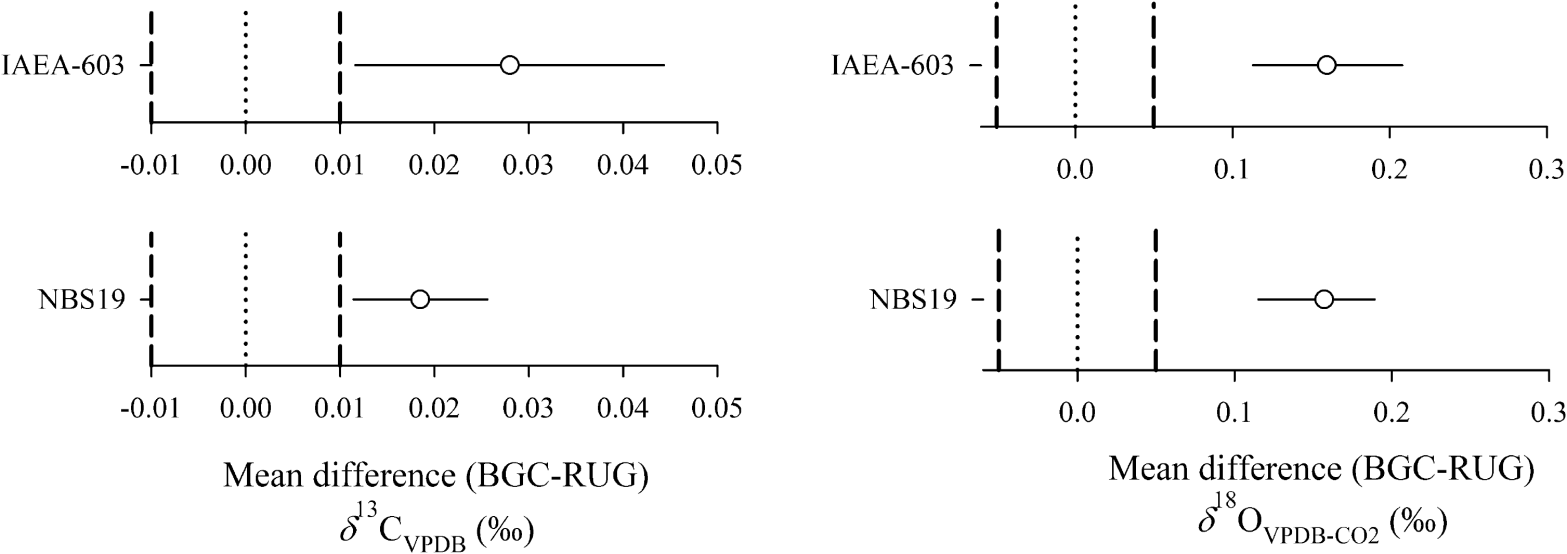
Assessing the equivalence of the stable isotope signatures from carbonate‐CO_2_ produced at BGC and RUG and measured at RUG based on two one‐sided tests (TOSTs). The data points indicate the mean difference between the datasets with associated 90% confidence intervals. The vertical dashed lines indicate the lower and upper boundaries as defined by the WMO‐GAW network compatibility goals (*δ* ^13^C_VPDB_: ±0.01‰; *δ* ^18^O_VPDB‐CO2_: 0.05‰). If the confidence intervals cross the boundaries, then the data is not equivalent within those boundaries. The zero point is indicated by the dotted line. If the confidence interval crosses the zero point, then the respective datasets from the two laboratories are not significantly different from each other.

The CO_2_ generated at RUG was analyzed at BGC together with the CO_2_ produced at BGC from the same material (Figure 2). The RM‐CO_2_ produced and analyzed at BGC was sent to RUG to be analyzed there together with CO_2_ from the same material produced at RUG (Figure 3). Due to the unconstrained scale contraction at RUG, only NBS 19 and IAEA‐603 data analyzed at RUG are shown here. The effects of the phosphoric acid reaction on the isotopic composition can be studied by producing carbonate RM CO_2_ gases in two laboratories and analyzing them in one laboratory on one instrument only.

Figure 2 shows the mean differences and 90% CIs of RM CO_2_ produced at BGC and RUG and measured at BGC. The TOST analysis was used to detect statistical differences. The *δ* ^13^C_VPDB_ values of the BGC‐produced CO_2_ are, on average, 0.004‰ more negative than the CO_2_ of the same RM produced at RUG. Only the RUG average USGS44 value is more positive than that of BGC. The average offset suggests a small, but systematic effect in the phosphoric acid reaction either at RUG or BGC. Such an offset can arise from the isotopic fractionation that occurs as CO_2_ gas is removed from the phosphoric acid and collected for example through incomplete extraction. This offset is comparable to previous inter‐comparisons where slightly better agreement (0.003‰) between participating laboratories was achieved [29]. No clear pattern is apparent from the *δ* ^13^C_VPDB_ results of the individual RM CO_2_. The *δ* ^13^C_VPDB_ RUG and BGC datasets of IAEA‐603, IAEA‐610, and USGS44 CO_2_ are neither equivalent within the WMO‐GAW network compatibility goals nor identical (Figure 2). The IAEA‐611, IAEA‐612, and MAR‐J1 datasets are statistically identical but not equivalent. Taken together, the average differences of all carbonate *δ* ^13^C syntheses from RUG and BGC (*n* = 91) are statistically indistinguishable from each other and at the same time equivalent within the WMO‐GAW network compatibility goals. The data show that the phosphoric acid reaction introduces a large enough variability that repeat syntheses are required to get a reliable mean value. Because the TOST procedure accounts for instances where only a few measurements have been made [43], at least 10 independent carbonate samples should be evolved to CO_2_ using separate phosphoric acid reactions. This is in line with the German industrial standard DIN38402‐71:2020‐10 [42] that suggests at least eight measurements per laboratory be made.

The RUG produced RM *δ* ^18^O_VPDB‐CO2_ data are, on average, 0.13‰ more negative than that produced by BGC (Figure 2). With an offset of 0.2‰, the IAEA‐611 and IAEA‐12 datasets stand out. None of the carbonate *δ* ^18^O_VPDB‐CO2_ datasets are equivalent within the WMO‐GAW network compatibility goals of 0.05‰ [13]. Strikingly, all of the datasets are statistically different from each other, which is likely due to differences in the production of CO_2_ via the phosphoric acid reaction, as discussed below.

The IAEA‐603 and NBS 19 CO_2_ produced at BGC was sent to RUG and measured there against locally produced CO_2_ from the same RMs (Figure 3). The average offset between the *δ* ^13^C_VPDB_ CO_2_ gases analyzed at RUG, but produced at BGC and RUG, is 0.02‰, with the BGC *δ* ^13^C_VPDB_ data more positive than the RUG *δ* ^13^C_VPDB_ data. The offset measured at RUG is larger, and of a different sign than the one reported for the CO_2_ gases analyzed at BGC‐IsoLab (Figure 2). This discrepancy can be caused by various effects. RUG produces different, fresh batches of CO_2_ gases for the measurements done at BGC‐IsoLab and for those done at RUG. This could indicate that repetitive phosphoric acid reactions at RUG over time may produce CO_2_ gases that are isotopically slightly heterogeneous. Another cause may be storage‐induced changes in the isotope signature of the BGC samples as these were stored for several months at RUG prior to measurement. In addition, the larger and “inverse” offset at RUG can also be explained by the fact that BGC gases are compared to freshly produced CO_2_ gases which are measured in the same measurement series. At BGC, the RUG‐produced CO_2_ is measured against a working standard which is calibrated against NBS 19, thereby averaging out any heterogeneity of the phosphoric acid production method of BGC. In contrast to the *δ* ^13^C_VPDB_ measurements at RUG, the *δ* ^18^O_VPDB‐CO2_ measurements at RUG show a very similar offset between the BGC and RUG datasets of 0.16‰ with more negative *δ* ^18^O_VPDB‐CO2_ CO_2_ from RUG compared to BGC *δ* ^18^O_VPDB‐CO2_ results (Figure 3). Here too, the BGC and RUG datasets are statistically different from each other as indicated by the TOST statistical test.

##### 3.2.1.1 Phosphoric Acid Reaction

The phosphoric acid reaction is central to high precision isotope measurements of CaCO_3_ (Equation 2):

(2) $${\text{CaCO}}_{3}+H_{3}{\mathrm{PO}}_{4}➔{\text{CaHPO}}_{4}+H_{2}O+{\mathrm{CO}}_{2}$$

Analytical conditions, such as reaction temperature [29], the water content (and its isotopic composition) of the phosphoric acid [20], CO_2_ yield, and grain size [53, 54], can affect the *δ* ^18^O isotopic composition of the evolved CO_2_ gas. The final isotope results of acid‐evolved CO_2_ are subject to uncertainties that are introduced by the phosphoric acid reaction and that of the isotope measurement. The continuous quality control measurements of pure CO_2_ gas at BGC show a long‐term standard deviation of 0.006‰ and 0.009‰ for *δ* ^13^C_VPDB_ and *δ* ^18^O_VPDB‐CO2_ measurements, respectively (Data S1). For the following consideration, the BGC and RUG IAEA‐603 data are used because this material underwent the most phosphoric acid syntheses in both laboratories (Table 1). The BGC IAEA‐603 CO_2_ has an average standard deviation of 0.01 and 0.04‰ for *δ* ^13^C_VPDB_ and *δ* ^18^O_VPDB‐CO2_, respectively (Table 1). This uncertainty incorporates that of the BGC instrumental long‐term precision above, and an independent uncertainty component introduced by the phosphoric acid reaction. When these two uncertainties are combined in squared form, then the phosphoric acid reaction at BGC adds an uncertainty of 0.008‰ and 0.039‰ to the mean IAEA‐603 *δ* ^13^C_VPDB_ and *δ* ^18^O_VPDB‐CO2_ values, respectively. The RUG produced IAEA‐603 CO_2_ that was analyzed at BGC has an average standard deviation of 0.01‰ and 0.07‰ for *δ* ^13^C_VPDB_ and *δ* ^18^O_VPDB‐CO2_ average data, respectively. Using the same approach as above, the RUG phosphoric acid reaction adds 0.008‰ and 0.069‰ of uncertainty to the mean *δ* ^13^C_VPDB_ and *δ* ^18^O_VPDB‐CO2_ values of the RUG produced IAEA‐603‐CO_2_ gas. This consideration indicates that both RUG and BGC phosphoric acid procedures add about the same uncertainty as the instrumental measurement to the IAEA‐603 *δ* ^13^C_VPDB_ values. In contrast, the uncertainty added by BGC and RUG phosphoric acid reactions to the final *δ* ^18^O_VPDB‐CO2_ CO_2_ values is a factor of ~5 and 8 greater than the instrumental uncertainty. This result supports previous findings that the oxygen isotopic composition of CO_2_ is very susceptible to the phosphoric acid reaction conditions [20, 29].

**FIGURE 4:**
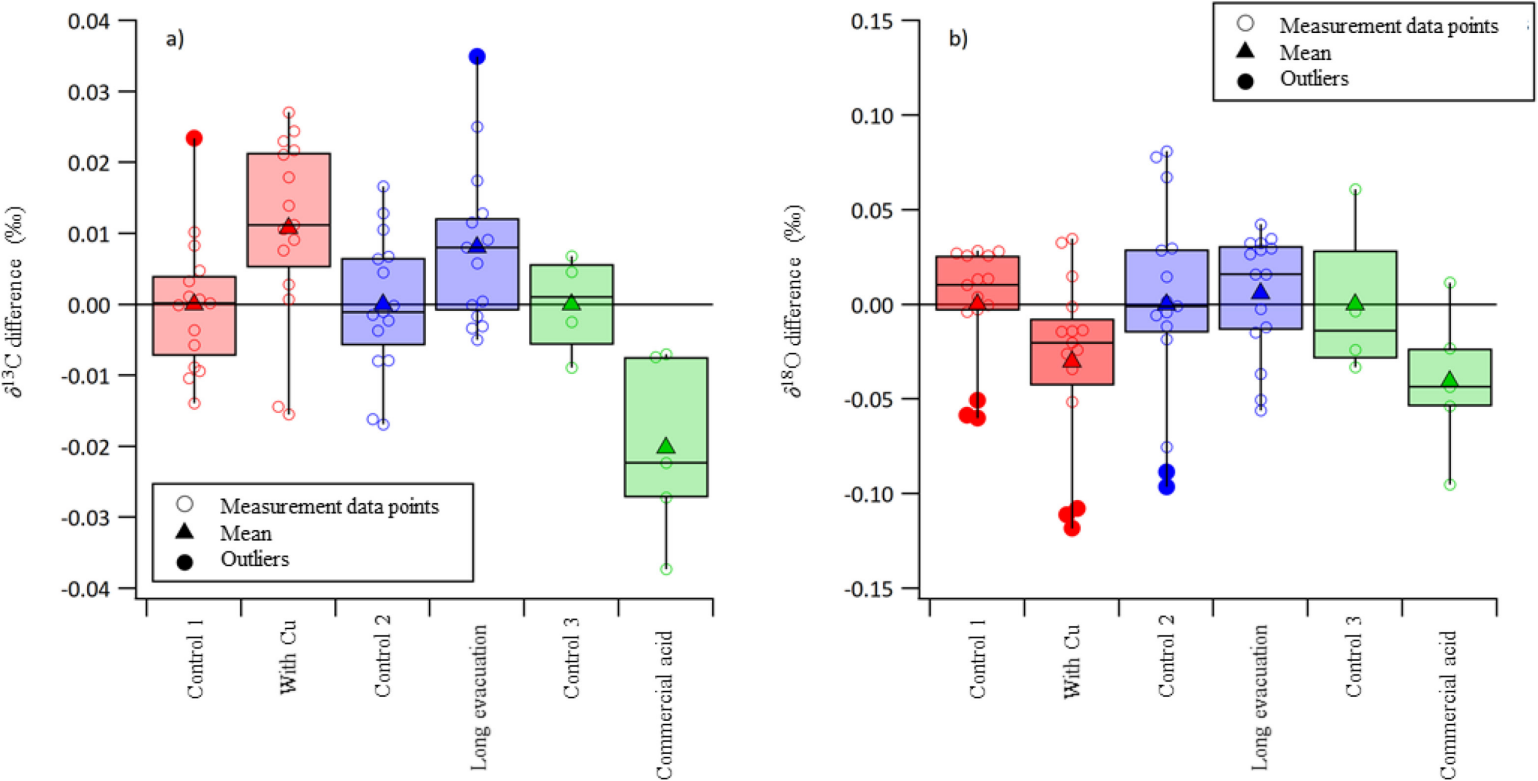
A series of three survey experiments conducted at RUG to investigate possible sources of uncertainty in the phosphoric acid reaction that may account for discrepancies observed between the BGC and the RUG *δ* ^13^C_VPDB_‐ and *δ* ^18^O_VPDB‐CO2_ data. The x‐axis represents the different tested conditions and the y‐axis represents the residuals (measurements—mean of the control). Controls represent the set of samples prepared using the regular preparation procedure and for every set of test condition a control set was prepared.

One concern is the temperature control of the phosphoric acid reaction. Its effect on the *δ* ^18^O_VPDB‐CO2_ values of NBS 19‐ and MAR‐J1‐CO_2_ has been quantified for the offline setup ARAMIS at BGC and is −0.04‰/°C [29, 30]. For calcite, values as low as −0.030‰/°C have been reported [55]. For a comprehensive review on the temperature dependence of the carbonate phosphoric acid reaction, we refer the reader to Kim et al. [56]. It is unlikely that the above calculated uncertainty components of the phosphoric acid reaction, 0.039‰ and 0.069‰, can be explained by reaction temperature fluctuations, as these would have to be in the order of ±0.5°C and ±0.8°C for BGC and RUG, respectively. However, both laboratories carefully control the reaction‐vessel temperature at 25 ± 0.1°C. At RUG, the temperature conductance of the reaction apparatus was tested (Figure 4). Because glass is a poor heat conductor, the reaction may reach a slightly elevated temperature which is dissipated only slowly. To check this hypothesis, 25 × 5 mm copper balls were thoroughly cleaned and placed together with the carbonate samples in the McCrea‐type flasks to increase the thermal mass within the reaction volume and reduce any potential temperature changes. A set of five such samples was prepared and compared against a set of another five samples prepared using the regular procedure in the same batch (control). Each sample was measured three times and all measurement results are represented in Figure 4. The samples prepared with Cu‐balls within the reaction volume show an offset of +0.01‰ and −0.02‰ in *δ* ^13^C and *δ* ^18^O values, respectively, compared to the control group. The reaction temperature does not have an influence on the resulting *δ* ^13^C values, which is in accordance with previous findings [29]. By adding copper balls into the reaction vessel, the heat capacity was increased, offsetting potential temperature build‐up during the reaction. The resulting lower temperature would cause the *δ* ^18^O values to be more positive [30, 57]. Therefore, the trend towards more negative *δ* ^18^O values cannot be explained by this experiment, and it is concluded that temperature variations cannot explain the *δ* ^18^O offset between the BGC and RUG phosphoric acid reactions.

Another cause for the added uncertainty in the *δ* ^18^O values is the water content of the phosphoric acid and subsequently the water available for equilibrium reactions with the CO_2_ during the reaction [20]. At BGC, the system is evacuated usually overnight to at least 1.6 × 10^−7^ mbar. Additionally, the reaction chamber of ARAMIS is made of gold‐coated copper, as a clean gold surface can be considered hydrophilic [58]. Wendeberg et al. [20] have shown that metal surfaces seem to be more suitable than the traditional glass surface for the reaction chamber of the phosphoric acid reaction. That, together with good repeatability (Table 1) over a long period of time (10 NBS 19 syntheses take 5 weeks) permits the conclusion that equilibrium reactions between water contamination and CO_2_ do not introduce the above‐discussed uncertainty in the *δ* ^18^O_VPDB‐CO2_ values of produced CO_2_ gas at BGC. To rule out the effect of dissolved gases in phosphoric acid and any adsorbed water in the carbonate materials or on glass surfaces, a set of five samples was evacuated extensively at RUG by submerging the McCrea‐type glass flasks in hot water (~80°C) for about 3 h while being evacuated and further evacuation of the samples overnight. This extended evacuation procedure was again compared to a control set (Figure 4). No significant difference is observed between the normal and extensively evacuated datasets and thus adsorbed water on surfaces or in the phosphoric acid cannot explain the observed discrepancies.

Wendeberg et al. [20] have shown that the oxygen isotopic composition of evolved CO_2_ is affected by an increase in water content of the phosphoric acid, i.e., a lower density acid. This dependence stops at a phosphoric acid density above 1.9 g/cm^3^ (at 25°C), which equates to phosphoric acid concentrations greater than 102% [20]. All three laboratories in this study prepare and use such high concentration phosphoric acid when producing CO_2_ for stable isotope analyses from carbonates. Nevertheless, RUG tested the susceptibility of the reaction to different batches of phosphoric acid. A set of five samples was prepared using commercially available 105% phosphoric acid (Merck, 398608‐1L, *ρ* = 1.9326 g/cm^3^). Again, this set of five measurements was compared against a control set, made with a freshly prepared batch of phosphoric acid (*ρ* = 1.9435 g/cm^3^). Unlike the previous two sets, here, every sample was measured just once. Surprisingly, the samples prepared using the commercially available acid showed a much larger spread in the *δ* ^13^C measurements than in the control set (or in any other of the tests), whereas the spread in the *δ* ^18^O measurements was similar (if not smaller) than in the controls. The difference between the two datasets cannot explain the *δ* ^18^O discrepancies we observe.

One of the key differences between the BGC and RUG procedures is the way the CO_2_ is collected. While at BGC the CO_2_ is passively frozen into the sample vial using liquid nitrogen until the pressure stabilizes, RUG uses a vacuum pump to gently extract the remaining CO_2_ from the phosphoric acid after the passive freezing process has led to a pressure of ~0.1 mbar. The difference in procedure likely affects the amount of CO_2_ remaining in the phosphoric acid and thus likely causes isotope fractionation effects of different magnitudes [57]. The equilibrium between CO_2_ remaining in the phosphoric acid, and that extracted cryogenically is further influenced by the amount of phosphoric acid and calcium carbonate used. Wendeberg et al. [20] have suggested that an intermediate carbonic acid molecule is involved in the phosphoric acid reaction. Applying a vacuum to further extract CO_2_ from the phosphoric acid will have an isotopic effect as more of the carbonic acid molecules dissociate into H_2_O and CO_2_, splitting off one oxygen atom as water and, therefore, changing the isotope composition of the other two oxygen atoms in the CO_2_. This explains why the isotope fractionation is primarily observed in the *δ* ^18^O values and not in the *δ* ^13^C values of the CO_2_ gas (Figures 1 and 2). Even under ideal vacuum conditions, the amount of water adhering to different apparatus material surfaces (glass vs. metal), facilitating an O‐atom exchange between CO_2_ and H_2_O [20] very likely also explains the *δ* ^18^O signature differences observed between the laboratories.

In conclusion, the interlaboratory comparability of *δ* ^13^C_VPDB_ values of the RMs is only good enough to meet WMO‐GAW network compatibility goals when all RUG and BGC data are averaged. This is in part due to very low phosphoric acid syntheses per RM, which affects the TOST statistic, but also due to the differences in how the syntheses are done. One surprising result is the impact different commercial phosphoric acids can have on the *δ* ^13^C_VPDB_‐CO_2_ values and this warrants further investigation in the future. The *δ* ^18^O_VPDB‐CO2_ results of the individual RMs from the two laboratories are neither identical nor do they meet WMO‐GAW network compatibility goals [13]. Because the VPDB scale realization currently depends on calcium carbonate primary and secondary RMs, our findings indicate that the phosphoric acid reaction parameters need to be further refined. Beyond temperature, reaction duration and water content (density) of phosphoric acid, other parameters such as apparatus material and design, amounts of calcite vs. phosphoric acid, phosphoric acid isotopic composition and supplier and CO_2_ extraction procedure may also have to be specified to achieve a higher interlaboratory comparability of results. Alternatively, one might look for ways to produce isotopically consistent CO_2_ (or CO) results from calcites, such as, for example, via pyrolysis [59]. Another way could be to link the *δ* ^18^O_VPDB‐CO2_ scale to the *δ* ^18^O_VSMOW‐SLAP_ scale as proposed by Aerts‐Bijma et al. [60].

#### 3.2.2 BGC–IAEA Comparison

**FIGURE 5:**
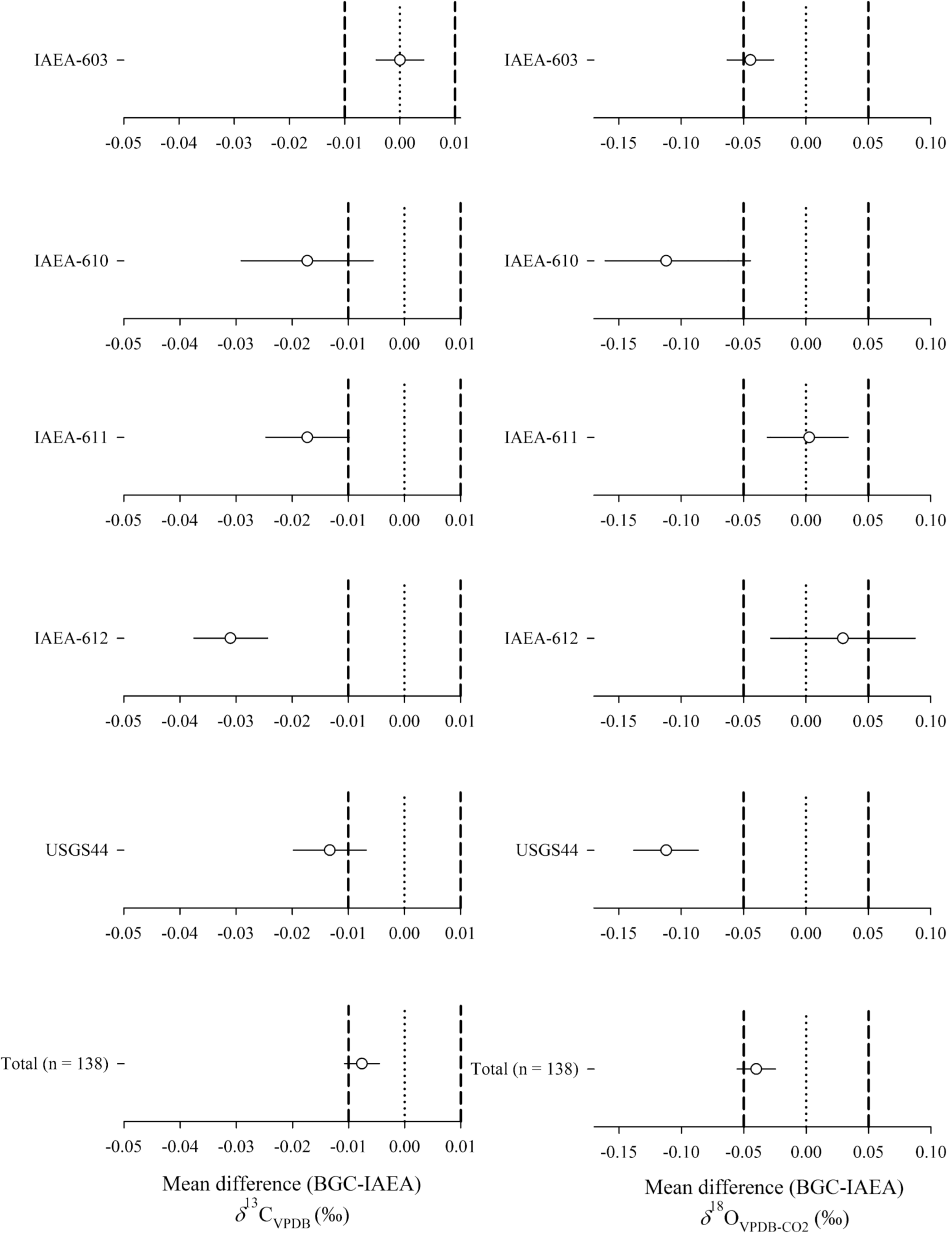
Two one‐sided tests (TOSTs) assessing the mean differences of the stable isotope signatures from carbonate‐CO_2_ produced and analyzed at BGC and IAEA. The data points indicate the mean difference between datasets with associated 90% confidence intervals. The vertical dashed lines indicate the lower and upper boundaries as defined by the WMO‐GAW network compatibility goals (*δ* ^13^C_VPDB_: ±0.01‰; *δ* ^18^O_VPDB_: 0.05‰). For the bottom two graphs all datasets are normalized to zero by subtracting each individual measurement by the mean of all measurements of one RM. If the confidence intervals cross the boundaries, then the data is not equivalent within those boundaries. The zero point is indicated by the dotted line. If the confidence interval crosses the zero point, then the respective means are considered not different.

IAEA and BGC produced independent isotope values for IAEA‐603, ‐610, ‐611, ‐612, and USGS44, with separate phosphoric acid reactions and instrumental measurements. In this section, we compare the interlaboratory comparability of these measurements. Figure 5 shows the mean differences and TOST analyses of the BGC and IAEA datasets of IAEA‐603, ‐610, ‐611, ‐612, and USGS44. The IAEA *δ* ^13^C_VPDB_ datasets of the studied RMs are on average 0.01‰ more negative than those of BGC. This offset is slightly larger than that reported for the BGC and RUG datasets and indicates that the independent phosphoric acid reactions of both laboratories have a small influence on the interlaboratory comparability of the datasets. An instrumental source for the offset seems unlikely due to the good agreement of NIST pure CO_2_ RM measurements between the two laboratories (Table 2), as discussed below. Only the IAEA‐603 *δ* ^13^C_VPDB_ dataset is equivalent within the GAW‐WMO network compatibility goal of 0.01‰ [13] owing to the large number of replicates that were done in both laboratories (Table 1). Additionally, the IAEA‐603 datasets from BGC and IAEA are statistically indistinguishable from each other. All other *δ* ^13^C_VPDB_ datasets are statistically different from each other and are not equivalent to the WMO‐GAW network compatibility goal. One striking observation of the laboratory comparison is that the IAEA and BGC dataset offsets increase with increasingly more negative isotope values from IAEA‐603 to ‐612, from no offset at all, to an offset of 0.03‰ (Figure 5). The USGS44 dataset does not follow this trend. The increasing offsets between the IAEA and BGC datasets could be a result of one of the two laboratories either over‐ or underestimating their instrumental scale correction. This was tested by both laboratories analyzing well‐known, pure CO_2_ gas RMs from the National Institute of Standards and Technology (NIST, [32], Table 2). BGC also analyzed pure CO_2_ gas RMs from the National Institute for Environmental Studies (NIES, [29] and references therein). The relatively low number of NIST RM replicate measurements by IAEA and BGC is due to the scarcity of these RM CO_2_ gases. Nevertheless, where both laboratories analyzed the same material (RM 8562 and RM8563), the agreement of the measured *δ* ^13^C_VPDB_ values is very good. In particular, the excellent agreement between the IAEA and BGC measurements and the literature value of RM8563 with its very negative *δ* ^13^C isotopic composition leads to the conclusion that the scale contraction corrections applied by both laboratories are correct (Table 2). The conclusion is supported by the better agreement between the measurements of USGS44 by the two laboratories as compared to IAEA‐612 (Figure 5). If scale contraction were the cause for the increasing offset exhibited by the IAEA RMs, then the USGS44 *δ* ^13^C measurements of BGC and IAEA would have to be offset even further from each other. The increasing offset between the IAEA and BGC IAEA‐610, ‐611, and ‐612 remains unclear for now and warrants further study.

**TABLE 2:** Pure CO_2_ RMs analyzed at BGC and IAEA. The uncertainty given is the standard deviation of the mean. The lab name in parenthesis indicates the analyzing laboratory. The literature values for NARCIS I and II are from Brand et al. [10, 29], and the values for the NIST RMs are from ([32]; Table 10‐D).

Sample description	Measurements		n	Literature values	
	*δ* ^13^C_VPDB_ [‰]	*δ* ^18^O_VPDB‐CO2_ [‰]		*δ* ^13^C_VPDB_ [‰]	*δ* ^18^O_VPDB‐CO2_ [‰]
NARCIS‐I (BGC)	−8.625 ± 0.003	−0.8 ± 0.01	4	−8.55 ± 0.02	−0.7 ± 0.06
NARCIS‐II (BGC)	1.896 ± 0.006	−2.71 ± 0.02	7	1.923 ± 0.01	−2.62 ± 0.014
RM8562 (BGC)	−3.740 ± 0.011	−18.66 ± 0.02	4	−3.72 ± 0.04	−18.56 ± 0.09
RM8562 (IAEA)	−3.767 ± 0.003	−18.44 ± 0.01	2		
RM8563 (BGC)	−41.570 ± 0.001	−33.82 ± 0.01	3	−41.57 ± 0.09	−33.76 ± 0.08
RM8563 (IAEA)	−41.571 ± 0.006	−33.58 ± 0.02	2		
RM8564 (BGC)	−10.472 ± 0.002	−10.09 ± 0.01	3	−10.45 ± 0.02	−10.06 ± 0.05

In the *δ* ^18^O datasets, only the IAEA‐611 from BGC and IAEA are equivalent within the GAW‐WMO network compatibility goal of 0.05‰ [13], and they are also statistically identical (Figure 5). The IAEA‐612 IAEA and BGC datasets are statistically indistinguishable from each other, but not equivalent within the WMO‐GAW boundaries. The largest deviation is observed between the IAEA‐610 and USGS44 datasets from BGC and IAEA. These two RMs have the most ^18^O depleted signal (Table 1). Additionally, the offsets between the *δ* ^18^O_VPDB‐CO2_ measurements of RM8562 and 8563 are both greater than 0.2‰ (Table 2). It is unlikely that the observed offsets in the *δ* ^18^O_VPDB‐CO2_ measurements of the carbonate RMs and the NIST CO_2_ gases are a result of different or incorrect scale contraction corrections. The eta correction factors of the BGC and IAEA instruments are so close together, that scale contraction alone would result in an offset of only 0.001‰ when analyzing a fictitious sample with a δ^18^O isotope value of −30 against NBS 19 (*δ* ^18^O_VPDB‐CO2_: −2.20‰).

Again, the phosphoric acid reaction offers the most likely explanation for the large offset between the IAEA‐610 and USGS44 *δ* ^18^O_VPDB‐CO2_ datasets (Figure 5). This assumption is supported by the variability of the offsets of the RM *δ* ^18^O_VPDB‐CO2_ measurements between BGC and IAEA. Furthermore, because the RUG *δ* ^18^O_VPDB‐CO2_ data are, on average, more negative than those of BGC, and IAEA *δ* ^18^O_VPDB‐CO2_ data are more positive than BGC, a direct comparison of the *δ* ^18^O_VPDB‐CO2_ data from both labs reveals that the phosphoric acid reaction introduces an even larger lab‐to‐lab offset between IAEA and RUG. Besides the differences in the used apparatuses, a possible explanation is the different ways CO_2_ is extracted from the phosphoric acid in the three laboratories. As discussed above, RUG supports the cryogenic extraction of CO_2_ with a vacuum pump. BGC extracts cryogenically only. In contrast, the IAEA expands the CO_2_ into an evacuated sample vessel until equilibrium is reached. However, this hypothesis requires experimental proof.

We note that the discrepancies in the *δ* ^18^O data between BGC and IAEA may also be explained by the use of different batches of materials in the respective laboratories, as the oxygen isotopic composition of IAEA‐610, ‐611, ‐612, and USGS44 can be affected by atmospheric moisture [25, 26]. The materials used at BGC and IAEA may indeed suffer from atmospheric contamination. However, both laboratories are aware of this problem and take precautions to avoid such contamination.

Applying the phosphoric acid reaction to the value assignment of primary and secondary carbonate RMs for the δ^13^C_VPDB_ scale is a time‐ and resource‐consuming process, explaining the relatively small datasets that we compare here. Nevertheless, the data and the above discussion suggest that the study of the phosphoric acid reaction parameters, and their standardization, is crucial to reduce lab‐to‐lab variability in the future.

### 3.3 VPDB Scale(s) Evaluated

The *δ* ^13^C_VPDB_ scale is defined by the RM NBS 19 with a consensus *δ* ^13^C_VPDB_ value of 1.95‰ exactly. The RMs IAEA‐603, ‐610, ‐611, ‐612, and USGS44 are secondary scale anchors with values that were produced in different laboratories in response to the instability of LSVEC [21, 25, 26]. The aim of this study is to verify previously published values, particularly the *δ* ^13^C_VPDB_ value of USGS44, and to make sure that all secondary carbonate RMs can be combined to realize the *δ* ^13^C_VPDB_ scale for the purpose of 2‐ or multipoint calibrations [61, 62]. To do this, the values associated with the phosphoric acid reactions from all laboratories are taken into account even though they do contribute to interlaboratory variability of the data. However, the average values calculated in this section only consider measurements done at BGC and IAEA as the RUG instrument in its present clumped isotope configuration is not suitable for the 1‐point calibration due to its insufficient abundance sensitivity. The consensus values of the secondary RMs IAEA‐603, ‐610, ‐611, ‐612, and USGS44 are shown in Table 3. The IAEA‐603 and IAEA‐610 *δ* ^13^C_VPDB_ values are identical to the published values [21, 26]. The IAEA‐611 *δ* ^13^C_VPDB_ value is 0.01‰ more negative, and the IAEA‐612 value is 0.02‰ more negative, while the USGS44 value is 0.01‰ less negative than the respective previously published literature values [25, 26]. Qi et al. [25] reported two values for USGS44 on the *δ* ^13^C_VPDB_ scale: −42.08 ± 0.01‰ and −41.99 ± 0.01‰. The *δ* ^13^C_VPDB_ value of −42.07 ± 0.01‰ reported here points towards the former of the two Qi et al. [25] values to be correct. Within their measurement uncertainties, the values reported here are indistinguishable from the value assignments published by Assonov et al. [26] and Qi et al. [25]. To most readers, these differences between the values of this study, and those reported by Assonov et al. [26] and Qi et al. [25] may seem very small. however, particularly when analyzing samples which are very ^13^C depleted, for example, biogenic methane with a global mean value of −62‰ [48] such small differences can play a large role. The 0.02‰ difference between the IAEA‐612 literature *δ* ^13^C_VPDB_ value and the value calculated in this study would cause a 0.03‰ difference in results for samples with an isotope value of biogenic methane. Even such a small offset makes achieving the WMO‐GAW network compatibility goal of 0.02‰ for *δ* ^13^C‐CH_4_ measurements impossible [13].

**TABLE 3:** Average *δ* ^13^C_VPDB_ and *δ* ^18^O_VPDB‐CO2_ values and standard uncertainties calculated from the BGC and RUG data (measured at BGC) and the IAEA data for calcium carbonate RMs together with previously published literature values.

Reference material	Consensus values (this study)		Literature values		Literature
	*δ* ^13^C_VPDB_ [‰]	*δ* ^18^O_VPDB‐CO2_ [‰]	*δ* ^13^C_VPDB_ [‰]	*δ* ^18^O_VPDB‐CO2_ [‰]	
IAEA‐603	2.462 ± 0.011	−2.39 ± 0.07	2.46 ± 0.01	−2.37 ± 0.04	Assonov et al. [21]
IAEA‐610	−9.114 ± 0.011	−18.87 ± 0.09	−9.11 ± 0.01	−18.83 ± 0.04	Assonov et al. [26]
IAEA‐611	−30.815 ± 0.011	−4.26 ± 0.08	−30.80 ± 0.01	−4.22 ± 0.05	Assonov et al. [26]
IAEA‐612	−36.739 ± 0.020	−12.09 ± 0.03	−36.72 ± 0.02	−12.08 ± 0.06	Assonov et al. [26]
USGS44	−42.073 ± 0.015	−15.71 ± 0.08	−42.08 ± 0.01	−15.75 ± 0.07	Qi et al. [25]

This dataset provides an independent assessment of the IAEA‐610, ‐611, and ‐612 and USGS44 RM values providing a harmonized set of RMs that can be used to realize the *δ* ^13^C_VPDB_ scale. With these sets of RMs, the *δ* ^13^C_VPDB_ scale can be realized with a high level of confidence between the isotopic values of NBS 19 (1.95‰) and USGS44 (−42.07‰). Efforts are underway to extend the range of the *δ* ^13^C_VPDB_ scale that is covered by well‐characterized RMs.

Except for USGS44, the average *δ* ^18^O_VPDB‐CO2_ values for IAEA‐603, ‐610, ‐611, and ‐612 are more negative, but not significantly so, given the uncertainties, than previously reported values. The *δ* ^18^O_VPDB‐CO2_ value of USGS44 is 0.04‰ more positive, and 0.05‰ negative, than the values reported by Qi et al. [25] (*δ* ^18^O_VPDB‐CO2_: −15.75 ± 0.07‰ and −15.657 ± 0.02‰). In addition, the associated uncertainties of the values reported here are higher than those previously reported, making the difference from the reported values not statistically significant. The larger uncertainty of our values is explained by the fact that the phosphoric acid reactions done at three different laboratories contribute to the error. Our findings show that the phosphoric acid reaction does not seem to be fit for purpose to produce *δ* ^18^O_VPDB‐CO2_ values for carbonates with a high enough precision and accuracy to meet GAW‐WMO guidelines [13].

One way to evade this problem is for just one laboratory to produce RM values, as is the case for isotopes of CO_2_ in air measurements for the GAW‐WMO community [30, 52]. However, such a solution comes with significant drawbacks in regard to the long‐term stability of the scale; for example, if such a laboratory loses funding or suffers from crucial instrumental breakdowns. Alternatively, linking the *δ* ^18^O_VPDB‐CO2_ to the *δ* ^18^O_VSMOW_ scales via equilibration techniques, or pyrolysis may also be a viable option [59, 60]. While the reported *δ* ^18^O_VPDB‐CO2_ are not intended to serve as RM values, they may still be useful for some applications and inform ongoing efforts to produce *δ* ^18^O_VPDB‐CO2_ RMs.

#### 3.3.1 Linking the VPDB and VPDB‐LSVEC Scales

The IAEA experts meeting held in Vienna in January 2024 decided to endorse both the VPDB and VPDB‐LSVEC *δ* ^13^C scales [15]. The difference between the scales is that the *δ* ^13^C_VPDB_ scale is defined by NBS 19 alone while the *δ* ^13^C_VPDB‐LSVEC_ scale is defined by both NBS 19 and LSVEC, the latter with a consensus value of −46.60‰ [16]. This decision was motivated by the fact that the VPDB‐LSVEC scale is now well established in diverse fields of science and industry, particularly because of the availability of large amounts of different RMs, among which are materials that have been value assigned on the VPDB‐LSVEC scale only [10, 63, 64]. Furthermore, the implementation of the *δ* ^13^C_VPDB‐LSVEC_ scale has led to much better compatibility of results from different laboratories because it has enabled laboratories to use isotopically well‐defined RMs for recommended multipoint data calibration [61].

One easy way to transfer RM and sample values from the *δ* ^13^C_VPDB_ to the *δ* ^13^C_VPDB‐LSVEC_ scale and vice versa is to use a transfer equation such as the one that Hélie et al. [27] have proposed. They have shown that the *δ* ^13^C_VPDB_ (realized by IAEA‐603, ‐610, ‐611, and ‐612) and VPDB‐LSVEC (realized by NBS 19 and USGS44) scales diverge as they move away from the “0‰” point. At the value of USGS44 (−42.21 ± 0.05‰ on the *δ* ^13^C_VPDB‐LSVEC_ scale), an isotopic difference of 0.18‰ is reported [27] which is similar to the offset of 0.12‰ reported by Qi et al. [25]. With small changes to one or more RM values on the *δ* ^13^C_VPDB_ scale, the transfer equation from *δ* ^13^C_VPDB_ to *δ* ^13^C_VPDB‐LSVEC_ may need to be adjusted, or at least its applicability needs to be checked. Table 4 shows EA‐IRMS measurements of available RMs at BGC that were value assigned on the *δ* ^13^C_VPDB_ scale by analyzing them against NBS 19 (+1.95‰), with USGS44 (−42.07 ± 0.01) as the second anchor. The measured *δ* ^13^C_VPDB_ values agree remarkably well (average 0.02‰) with previously published *δ* ^13^C_VPDB_ values except IAEA‐CO‐8 and IAEA CO‐9. Both values were assigned in the mid‐90s [65, 66], and instrumental considerations, for example scale contraction corrections that have been introduced since then, merit re‐evaluation of these older value assignments. Table 4 also provides the first *δ* ^13^C_VPDB_ measurements of the sugar RMs introduced by Chartrand et al. [63] which previously only had values on the *δ* ^13^C_VPDB‐LSVEC_ scale.

**TABLE 4:** EA‐IRMS measurements of available RMs at BGC with values on the *δ* ^13^C_VPDB_ scale, scaled using NBS 19 and USGS44 (bold). The measured *δ* ^13^C_VPDB_ values are transferred to the *δ* ^13^C_VPDB‐LSVEC_ scale using the equation published by [27].

Sample description	This study [*δ* ^13^C_VPDB_]	*n*	Literature values [*δ* ^13^C_VPDB_]	Literature	Re‐calculated^a^	Literature values [*δ* ^13^C_VPDB‐LSVEC_]	Literature
					[*δ* ^13^C_VPDB‐LSVEC_]		
BEET‐1	−25.94 ± 0.04	12	N/A		−26.05 ± 0.05	−26.02 ± 0.07	Chartrand et al. [63]
FRUT‐1	−10.99 ± 0.05	14	N/A		−11.03 ± 0.05	−10.98 ± 0.05	Chartrand et al. [63]
GALT‐1	−21.32 ± 0.05	11	N/A		−21.41 ± 0.06	−21.41 ± 0.06	Chartrand et al. [63]
IAEA‐600	−27.64 ± 0.02	8	−27.68^c^	Qi et al. [25]	−27.76 ± 0.04	−27.77 ± 0.04	Coplen et al. [16]
IAEA‐601	−28.68 ± 0.033	8	−28.72^c^	Qi et al. [25]	−28.81 ± 0.05	−28.81 ± 0.04	Coplen et al. [16]
IAEA‐603	2.47 ± 0.06	13	2.46 ± 0.01	Assonov et al. [21]	2.48 ± 0.06	N/A	
IAEA‐610	−9.13 ± 0.06	22	−9.11 ± 0.01	Assonov et al. [26]	−9.17 ± 0.06	N/A	
IAEA‐611	−30.82 ± 0.07	31	−30.80 ± 0.01	Assonov et al. [26]	−30.95 ± 0.08	N/A	
IAEA‐612	−36.75 ± 0.05	66	−36.72 ± 0.02	Assonov et al. [26]	−36.91 ± 0.07	N/A	
IAEA‐CH6	−10.48 ± 0.07	22	−10.43 ± 0.13	Gonfiantini et al. [65]	−10.52 ± 0.07	−10.45 ± 0.03	Coplen et al. [16]
IAEA‐CH7	−32.04 ± 0.04	7	−31.83 ± 0.11	Gonfiantini et al. [65]	−32.18 ± 0.05	−32.15 ± 0.05	Coplen et al. [16]
IAEA‐CO‐1	2.43 ± 0.06	12	2.48 ± 0.03	Stichler, [66]	2.44 ± 0.06	2.49 ± 0.03	Coplen et al. [16]
IAEA‐CO‐8	−5.86 ± 0.07	10	−5.75 ± 0.06	Stichler, [66]	−5.88 ± 0.07	−5.76 ± 0.03	Coplen et al. [16]
IAEA‐CO‐9	−47.30 ± 0.11	8	−47.12 ± 0.15	Stichler, [66]	−47.5 ± 0.12	−47.32 ± 0.05	Coplen et al. [16]
MAR‐J1	1.99 ± 0.06	21	1.96 ± 0.01	Brand et al. [29]	−2.00 ± 0.06	N/A	
**NBS 19**	**1.95**	62	1.95	Hut [17]	1.95^b^	1.95	Coplen et al. [16]
NBS22	−29.91 ± 0.05	25	−29.99 ± 0.05	Stalker et al. [67]	−30.04 ± 0.06	−30.03 ± 0.05	Coplen et al. [16]
USGS40	−26.26 ± 0.02	6	−26.24 ± 0.07	Qi et al. [68]	−26.37 ± 0.04	−26.39 ± 0.04	Coplen et al. [16]
**USGS44**	**−42.07 ± 0.06**	21	−42.08 ± 0.01	Qi et al. [25]	−42.25 ± 0.07	−42.21 ± 0.05	Qi et al. [25]

^a^ Values are recalculated using the equation published by [27].

^b^ The NBS 19 value +1.95 exactly is part of the definition of the δ^13^C_VPDB‐LSVEC_ scale. The transfer equation published by Hélie et al. [27] gives a value of +1.961 ± 0.08‰ for NBS 19 on the δ^13^C_VPDB‐LSVEC_ scale when the δ^13^C_VPDB_ defining value of +1.95‰ is used.

^c^ Qi et al. [25] give no uncertainty for these values. They scale the values vs. USGS44 (−42.08 ± 0.05) so that an uncertainty of at least 0.05‰ can be assumed.

The EA‐IRMS measurement values are transferred from the *δ* ^13^C_VPDB_ to the *δ* ^13^C_VPDB‐LSVEC_ scale using the linear equation published by Helier et al. [27]. The re‐calculated *δ* ^13^C_VPDB‐LSVEC_ values shown in Table 4 also agree very well with literature values. The average offset between the re‐calculated and literature *δ* ^13^C_VPDB‐LSVEC_ values is 0.02‰ and thus well within the uncertainty budget of the measurements conducted for this study and that of literature values (Table 4). Here too, the largest offsets are observed for IAEA‐CO‐8 and IAEA‐CO‐9, again suggesting that the older value assignments should be re‐evaluated. The generally good agreement between the measurement and the literature *δ* ^13^C_VPDB‐LSVEC_ values shows the viability of the transfer equation published by Hélie et al. [27]. The slightly different values that are measured and used here for IAEA‐610, ‐611, and 612 RMs are too small to invalidate the use of the transfer equation [27]. Table 4 gives values for IAEA‐610, ‐611, and ‐612 on the *δ* ^13^C_VPDB‐LSVEC_ scale that are 0.03‰, 0.13‰, and 0.15‰ more negative than those on the *δ* ^13^C_VPDB_ scale. These calculated offsets fit well with previously published offsets between the two scales [25, 27]. To our knowledge, these are the first published values for IAEA‐610, ‐611, and ‐612 on the *δ* ^13^C_VPDB‐LSVEC_ scale, and these values can be used by colleagues who wish to use these standards for EA‐IRMS measurements. We stress that the recalculated *δ* ^13^C_VPDB‐LSVEC_ values of the RMs shown in Table 4 need to be verified by actual measurements, as the transfer function introduces uncertainties that can lead to incorrect values [27]. Indeed, when applying the literature value of NBS 19 (*δ* ^13^C_VPDB_: 1.95‰) to the function, the result on the *δ* ^13^C_VPDB‐LSVEC_ scale is 1.961‰. This is not correct. The value of NBS 19 is +1.95‰ exactly, on both scales [15, 16]. Thus, previously published *δ* ^13^C_VPDB‐LSVEC_ standard values that have been verified by measurements should be used (e.g., [10]).

## 4 Conclusions

This study re‐examines the assignments of IAEA‐610, ‐611, ‐612, and USGS44 *δ* ^13^C_VPDB_ values with the aim of providing a unified set of RMs that can be combined to realize the *δ* ^13^C_VPDB_ scale. When the measurements from IAEA and BGC were taken together, the *δ* ^13^C_VPDB_ values of IAEA‐603 and ‐610 are identical to those published previously [21, 26]. The *δ* ^13^C_VPDB_ values reported in this study for IAEA‐611 and ‐612 RMs of −30.815 ± 0.011‰ and −36.739 ± 0.020‰, respectively, are slightly more negative than the previously published ones [26]. The measured USGS44 *δ* ^13^C_VPDB_ value is −42.073 ± 0.015‰, slightly more positive than the previously published value of −42.08 ± 0.01 [25]. These values are not statistically different from the previously published values. With these values, the RMs can be combined to realize the *δ* ^13^C_VPDB_ scale with a high degree of accuracy. This is particularly important in fields where a high degree of interlaboratory compatibility is required, and where very ^13^C depleted samples are analyzed, as is the case for measurements of atmospheric methane within the WMO‐GAW network [48, 69]. Whether the reported *δ* ^13^C_VPDB_ values will be adopted as new best measurements will be up to the IAEA experts' groups. Regardless of their decision, the authors of this study suggest that further measurements be made of these and other RMs to improve the data foundation on which the *δ* ^13^C_VPDB_ and *δ* ^13^C_VPDB‐LSVEC_ scales are based. The evolution of the NBS22 *δ* ^13^C_VPDB_ (and *δ* ^13^C_VPDB‐LSVEC_) value is a good example of this process that leads to refined and accepted RM values [27].

As part of this work, the impacts of phosphoric acid reactions and the instrument‐specific scale contraction were studied. By analyzing CO_2_ produced at BGC and RUG from the same RM on the same instrument, this study confirms previous findings that the *δ* ^13^C_VPDB_ values of calcium carbonates are only slightly, but significantly affected by different reaction procedures. This is also true for the independent measurements done at IAEA. In contrast, producing CO_2_ gases at different laboratories with a homogeneous oxygen isotopic composition remains a challenge even where expert laboratories very carefully control their reaction parameters. This is apparent from the relatively large uncertainties associated with the *δ* ^18^O_VPDB‐CO2_ values of the analyzed RMs. The *δ* ^18^O_VPDB‐CO2_ values reported here for IAEA‐610, ‐611, ‐612, and USGS44 are −18.87 ± 0.09‰, −4.26 ± 0.08‰, −12.09 ± 0.03‰, and −15.71 ± 0.08‰, respectively (Table 3). We stress that these RMs are not intended to realize the *δ* ^18^O_VPDB‐CO2_ scale, and so far, a reliable second anchor for the *δ* ^18^O_VPDB‐CO2_ scale is woefully missing. We provide the values here as they may be useful to ongoing efforts to produce RMs for the *δ* ^18^O_VPDB‐CO2_ scale.

To improve the interlaboratory comparability of *δ* ^18^O_VPDB‐CO2_ values from calcium carbonate RMs, we suggest that the parameters of the phosphoric acid reaction, most prominently the way CO_2_ is extracted and collected, need to be further constrained. Beyond controlling reaction parameters such as reaction time, temperature, and phosphoric acid water content, the phosphoric acid reaction apparatus material and design may have to be unified, and a consensus CO_2_ extraction/collection protocol developed. RM‐producing laboratories could further improve interlaboratory comparability of *δ* ^18^O_VPDB‐CO2_ values of calcium carbonates by defining a phosphoric acid reaction protocol with narrow specifications for the reaction parameters. Other approaches such as linking the *δ* ^18^O_VPDB‐CO2_ and *δ* ^18^O_VSMOW‐SLAP_ scales, or producing CO_2_ from calcium carbonate with other methods are also promising [59, 60].

In the rare event where 1‐point calibrations need to be applied to measurements, for example, because only one primary RM is available, as was the case for this study, instrumental offsets that can lead to systematic errors need to be carefully evaluated. In this study, the instrument‐dependent cross‐contamination eta effect was carefully studied. Instruments from different manufacturers can have widely varying eta effects and, unfortunately, corrections are not always possible as was shown here.

Having two *δ* ^13^C scales is not ideal, and it is crucial to understand that it was never intended to implement a second scale with the introduction of LSVEC. Rather, the advent of LSVEC as a second scale anchor was intended to improve the interlaboratory comparability of *δ* ^13^C data, and this has been achieved. Only later, when the instability of LSVEC was discovered [22, 23], and colleagues realized that the value assignment of LSVEC was too negative, did the divergence of the *δ* ^13^C_VPDB_ and *δ* ^13^C_VPDB‐LSVEC_ scales become apparent [25, 27]. With IAEA‐610, ‐611, ‐612, and USGS44, enough secondary RMs with value assignments on the *δ* ^13^C_VPDB_ scale are available to perform 2‐ or even multipoint‐scale normalizations. Therefore, the *δ* ^13^C_VPDB‐LSVEC_ and *δ* ^13^C_VPDB_ are equally suitable for the purpose of producing accurate and precise *δ* ^13^C measurements. With the transfer equation published by [27], it is easy to re‐calculate values on either scale. Therefore, one could question whether both, the *δ* ^13^C_VPDB_ and δ^13^C_VPDB‐LSVEC_ scales, are required for *δ* ^13^C measurements. One of the primary reasons why the IAEA experts meeting has endorsed both the *δ* ^13^C_VPDB_ and *δ* ^13^C_VPDB‐LSVEC_ scales is the fact that the latter scale is now well established, and its discontinuation would necessitate a concerted effort to value assign a large quantity of RMs on the *δ* ^13^C_VPDB_ scale. Additionally, many standards used in specific fields such as forensics or food adulteration currently only have values assigned on the *δ* ^13^C_VPDB‐LSVEC_ scale, e.g., USGS43 (hair) and USGS82 (honey). Thus, the *δ* ^13^C_VPDB‐LSVEC_ scale remains critically important to measurements done in these fields. One of the drawbacks of keeping two scales will be that future RMs will require two values, one on the *δ* ^13^C_VPDB_, and the other on the *δ* ^13^C_VPDB‐LSVEC_ scale, as this study has done for IAEA‐610, ‐611, and ‐612 using the transfer equation published by Hélie et al. [27]. In addition, scientists will need to pay more attention to which scale is used to report their data, and it is suggested here that the minimum requirements for reporting stable isotope data are adhered to [34]. The discussion that was started by Qi et al. [25] and has recently been comprehensively reviewed by Dunn and Camin [70] around which of the two scales, the *δ* ^13^C_VPDB_‐ or *δ* ^13^C_VPDB‐LSVEC_ scale, to use has for now been laid to rest with both scales being endorsed by the IAEA experts meeting [15]. The data presented here support the decision of the IAEA experts meeting not to adopt the “VPDB2020” scale which was suggested by Assonov et al. [28]. Any future decisions made by the IUPAC‐CIAAW commission and the IAEA experts meeting regarding isotope scales should be supported by expert laboratories that continue to invest time and financial resources into the production of ever better characterized RMs with the aim of providing stable isotope scales to the ever‐growing isotope user communities.

## Author Contributions

**Heiko Moossen:** Conceptualization, Data Curation, Formal Analysis, Funding Acquisition, Invesigation, Methodology, Project Administration, Resources, Supervision, Writing ‐ Original Draft Preparation and Review and Editing. **Pharahilda M. Steur:** Data Curation, Formal Analysis, Investigation, Methodology, Validation, Writing ‐ Original Draft Preparation. **Federica Camin:** Data Curation, Funding Acquisition, Methodology, Project Administration, Resources, Supervision, Writing ‐ Original Draft Preparation. **Bor Krajnc:** Data Curation, Investigation, Formal Analysis, Methodology, Writing ‐ Original Draft Preparation. **Anett Enke:** Investigation **Heike Geilmann:** Investigation **Dipayan Paul:** Investigation, Visualization **Markus Lange:** Formal Analysis, Writing ‐ Original Draft Preparation **Isabell von Rein:** Investigation **Harro A. J. Meijer:** Conceptualization, Data Curation, Formal Analysis, Funding Acquisition, Methodology, Project Administration, Resources, Supervision, Validation, Writing ‐ Original Draft Preparation and Review and Editing.

## Conflicts of Interest

The authors declare no conflicts of interest.

## Supporting information


**Data S1:** Supporting information.

**Data S2:** Supporting information.

## Data Availability

The data that supports the findings of this study are available in the Supporting Information of this article.
