# Supplementary material for: How Well Do We Know VPDB—Part 2: Interlaboratory Assessment of Existing δ 13CVPDB Reference Materials
Source: Rapid Commun Mass Spectrom. 2025 Dec 4;40(4):e10171. doi: 10.1002/rcm.10171 (PMC12676516; doi:10.1002/rcm.10171)
Supplement: Supplementary file 1 — Data S1: Supporting information. [file RCM-40-e10171-s002.docx]

**Eta correction applied at BGC-Isolab**

At the BGC the sample/reference gas cross contamination (*η*-effect) is determined using idle-time experiments (Verkouteren et al., 2003) and corrected using the following equations:

*δ*_T_ = (*δ*_M_ – (*δ*_S_**η*))/(1-*η*) Eq. 1

*δ*_T_ is the drift- and *η*-corrected delta value of the sample, *δ*_M_ is the drift corrected delta value of the sample and *δ*_S_ is the drift corrected delta value of the working standard. *η* represents the cross-contamination factor that is determined by idle time experiments (figure 1) and calculated using equation 2:

*η* = (*δ*_M_ – *δ*_T_)/(*δ*_S_- *δ*_T_) Eq. 2


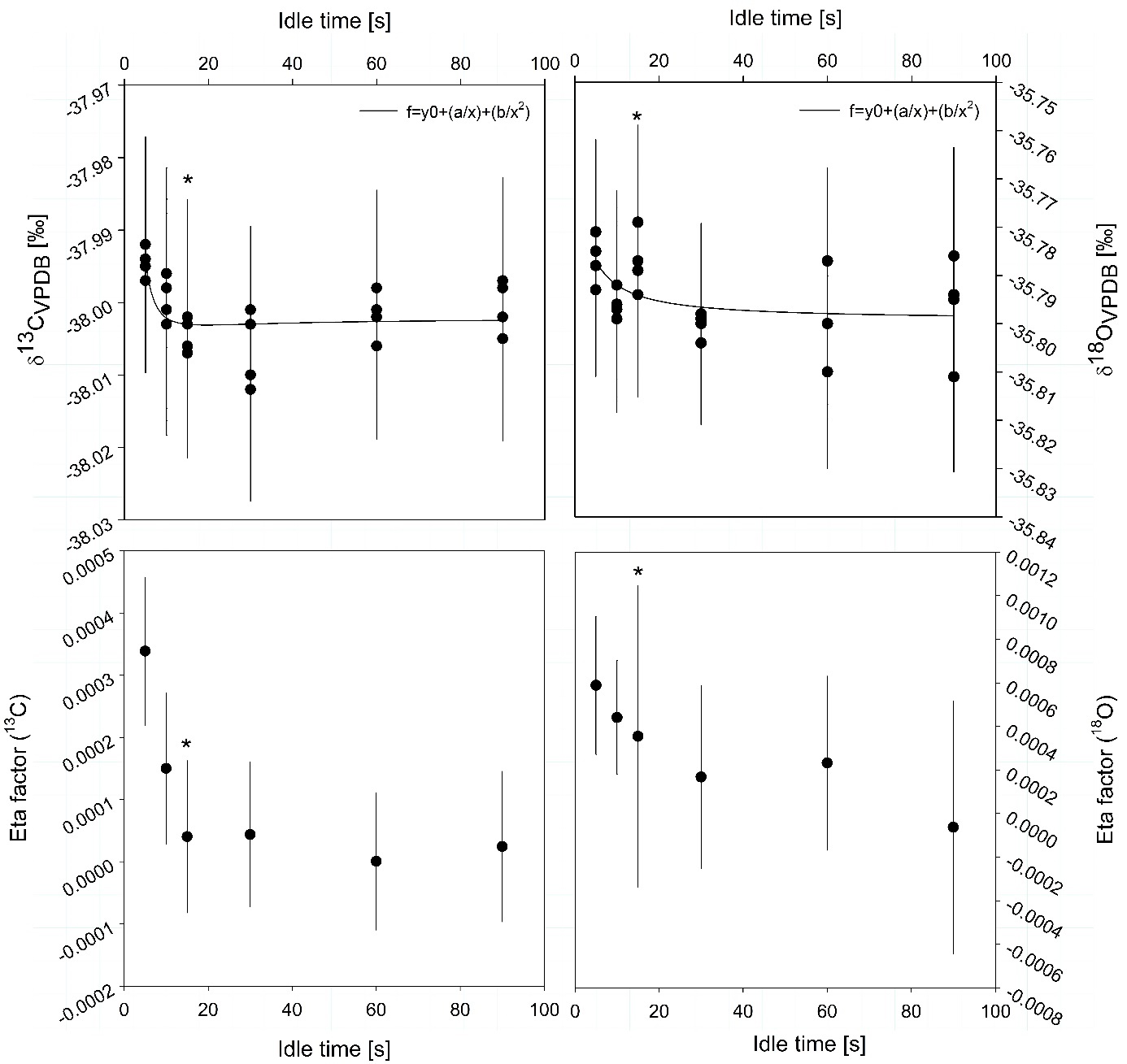


**Figure 1:** Experimental determination of the eta factor and its influence on measurements. Top: *δ*^13^C- and *δ*^18^O measurement dependence on idle time (asterisk indicates routine measurement conditions at an idle time of 15 s). Bottom: Eta factors of *δ*^13^C- and *δ*^18^O measurement for different idle times (asterix indicates routine measurement conditions at 15 s).

**Quality control measurements at BGC**

Figure 2 shows the quality control measurements between September 2021 and February 2025 of the CO_2_ gas “Simon” with *δ*^13^C_VPDB_ and *δ*^18^O_VPDB-CO2_ isotope values of -37.967 ± 0.006 ‰ and -35.793 ± 0.010 ‰, respectively.


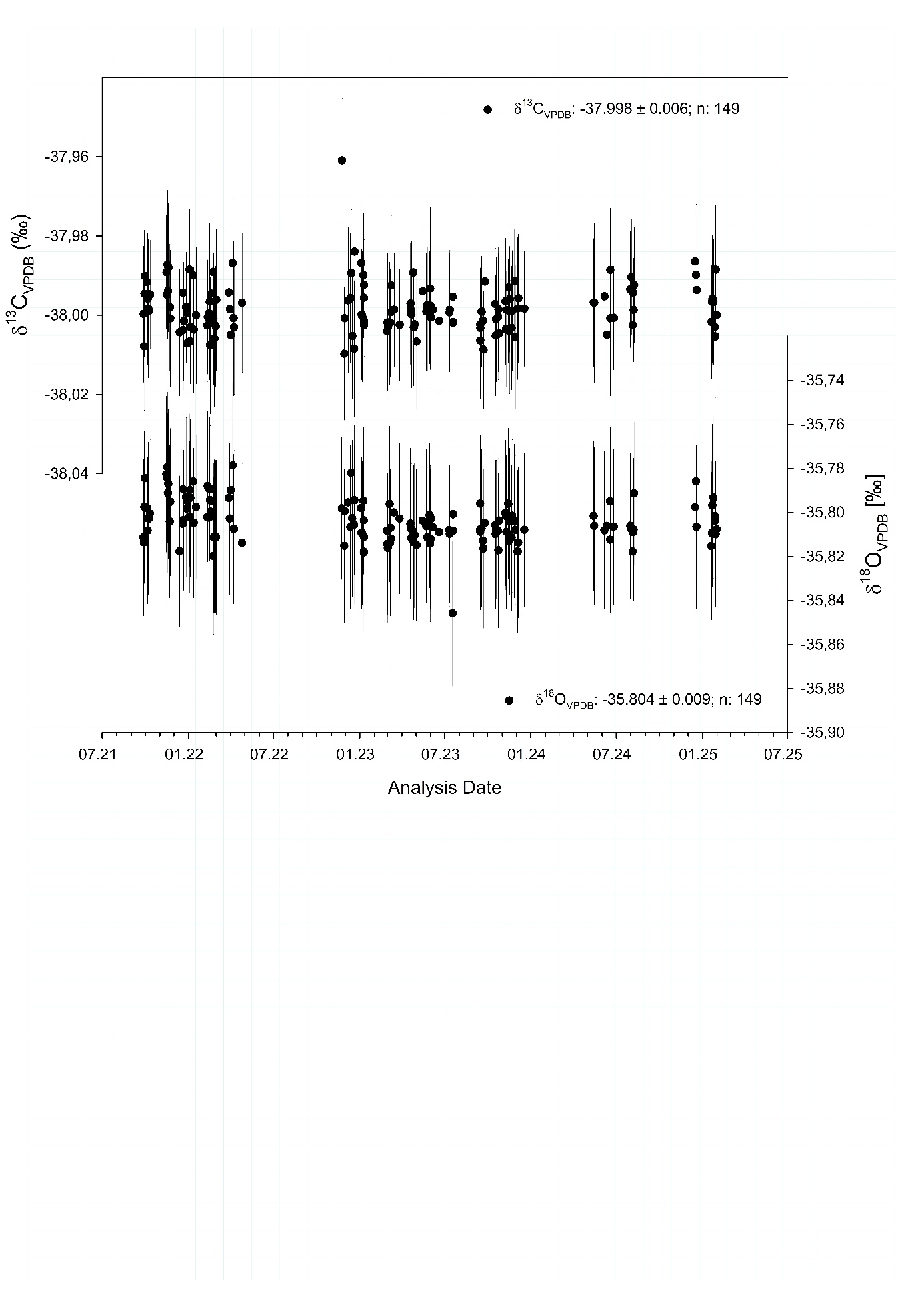


**Figure 2**: BGC-IsoLab quality control measurements of a pure CO_2_ gas “Simon” from September 2021 to January 2025. The average *δ*^13^C_VPDB_ and *δ*^18^O_VPDB_ values are -37.998 ± 0,006 ‰ and -35.804 ± 0.009 ‰, respectively. The average combined standard uncertainty of each individual measurement is 0.017 and 0.03 ‰ for *δ*^13^C_VPDB-CO2_ and *δ*^18^O_VPDB-CO2_ measurements, respectively.

**Uncertainty propagation of BGC-IsoLab measurements**

In the following the offline data handling and uncertainty propagation are detailed using a measurement sequence from the 25^th^ of February 2022. All uncertainty propagations follow the “Evaluation of measurement data - Guide to the expression of uncertainty in measurement” (JCGM, 2008). This can be verified by applying the “NIST uncertainty machine” to the example dataset below (https://uncertainty.nist.gov, Lafarge and Possolo, 2015). Table 1 shows a typical measurement sequence consisting of the working standard analyzed at the beginning and the end of the sequence, three sample gases, and the quality control gas “Simon”. All measurements in a daily sequence are done using one bellows filling of reference (monitoring) gas. In a first step a drift correction is applied to the working standard values. This drift is usually less than 0.01 for *δ*^13^C and *δ*^18^O values and is due to the reference gas drifting as it is isotopically depleted throughout the course of the sequence. Since this drift is not caused by either the working standard or sample gases it is not included in the uncertainty propagation.

Table 1: Typical measurement sequence of CO_2_ gases at the BGC-IsoLab. *δ*^13^C- and *δ*^18^O raw values are the ^17^O corrected values corrected against the reference gas. Each bellow filling is analyzed three times, each time with 20 change-over-valve cycles that measure the reference gas against sample gas. Thus, each bellow filling results in three values with associated standard deviations.

| Sample/Standard Name | δ^13^C (Raw) | δ^13^C (S.D.) | δ^18^O (Raw) | δ^18^O (S.D.) |
| --- | --- | --- | --- | --- |
| Working Standard CECILY2019a | -4.1138 | 0.0151 | -13.8598 | 0.0183 |
| CECILY2019a | -4.1077 | 0.0155 | -13.8474 | 0.0171 |
| CECILY2019a | -4.1077 | 0.0147 | -13.8566 | 0.0192 |
| NBS-19 20220298 Batch 791 | 1.8871 | 0.0111 | -2.3096 | 0.0187 |
| NBS-19 20220298 Batch 791 | 1.884 | 0.0114 | -2.312 | 0.0224 |
| NBS-19 20220298 Batch 791 | 1.8768 | 0.0181 | -2.3149 | 0.0165 |
| RM8562 20220299 CO2 NIST837*100126 | -3.8093 | 0.0088 | -18.7902 | 0.0192 |
| RM8562 20220299 CO2 NIST837*100126 | -3.8188 | 0.0187 | -18.8005 | 0.0175 |
| RM8562 20220299 CO2 NIST837*100126 | -3.8064 | 0.0134 | -18.7946 | 0.0096 |
| RM8564 20220297 CO2 NIST837*002093 | -10.533 | 0.0127 | -10.2001 | 0.0155 |
| RM8564 20220297 CO2 NIST837*002093 | -10.5449 | 0.0143 | -10.2015 | 0.0175 |
| RM8564 20220297 CO2 NIST837*002093 | -10.5334 | 0.0152 | -10.1935 | 0.0164 |
| Simon aliquot 20202178 | -38.0615 | 0.0139 | -35.9216 | 0.0229 |
| Simon aliquot 20202178 | -38.0748 | 0.0214 | -35.907 | 0.0162 |
| Simon aliquot 20202178 | -38.0676 | 0.0096 | -35.9168 | 0.0214 |
| Working Standard CECILY2019a | -4.1151 | 0.0133 | -13.8629 | 0.0255 |
| CECILY2019a | -4.1057 | 0.014 | -13.8521 | 0.0179 |
| CECILY2019a | -4.1121 | 0.0123 | -13.8662 | 0.0263 |

In the next step the drift corrected values are “Eta”-corrected (table 2) using the following equation:

*δ*_eta_ = (*δ*_M_ – (*δ*_S_**η*))/(1-*η*) Eq. 1

*δ*_eta_ is the eta corrected value, *δ*_M_ is the drift corrected raw individual delta value, *δ*_S_ is the average drift corrected value of the working gas “Cecily2019a”. The *η*-values are those determined in idle time experiments for an idle time of 15 seconds with values of 0.00004 ± 0.00012 and 0.000354 ± 0.00069 for *δ*^13^C and *δ*^18^O measurements, respectively.

Table 2: Eta corrected *δ*^13^C and *δ*^18^O measurements with associated propagated standard uncertainties.

| Sample/Standard Name | δ^13^C (eta corrected) | δ^13^C (S.D.) | δ^18^O (eta corrected) | δ^18^O (S.D.) |
| --- | --- | --- | --- | --- |
| Working Standard CECILY2019a | -4.1138 | 0.0151 | -13.8598 | 0.0228 |
| CECILY2019a | -4.1077 | 0.0155 | -13.8474 | 0.0218 |
| CECILY2019a | -4.1077 | 0.0147 | -13.8566 | 0.0235 |
| NBS-19 20220298 Batch 791 | 1.8876 | 0.0111 | -2.3044 | 0.0211 |
| NBS-19 20220298 Batch 791 | 1.8845 | 0.0114 | -2.3068 | 0.0244 |
| NBS-19 20220298 Batch 791 | 1.8773 | 0.0181 | -2.3097 | 0.0192 |
| RM8562 20220299 CO2 NIST837*100126 | -3.8088 | 0.0088 | -18.7897 | 0.0251 |
| RM8562 20220299 CO2 NIST837*100126 | -3.8183 | 0.0187 | -18.8000 | 0.0238 |
| RM8562 20220299 CO2 NIST837*100126 | -3.8059 | 0.0134 | -18.7941 | 0.0188 |
| RM8564 20220297 CO2 NIST837*002093 | -10.5325 | 0.0128 | -10.1954 | 0.0196 |
| RM8564 20220297 CO2 NIST837*002093 | -10.5444 | 0.0144 | -10.1968 | 0.0212 |
| RM8564 20220297 CO2 NIST837*002093 | -10.5329 | 0.0153 | -10.1888 | 0.0203 |
| Simon aliquot 20202178 | -38.0619 | 0.0147 | -35.9248 | 0.0352 |
| Simon aliquot 20202178 | -38.0752 | 0.0219 | -35.9102 | 0.0312 |
| Simon aliquot 20202178 | -38.0680 | 0.0107 | -35.9200 | 0.0342 |
| Working Standard CECILY2019a | -4.1139 | 0.0133 | -13.8571 | 0.0289 |
| CECILY2019a | -4.1045 | 0.0140 | -13.8463 | 0.0225 |
| CECILY2019a | -4.1109 | 0.0123 | -13.8604 | 0.0296 |

Uncertainties are propagated using the classical rules of uncertainty propagation (Table 4):

Table 4: Rules of uncertainty propagation for different types of calculation.

| **Type of calculation** | **Example** | **Uncertainty (s)** |
| --- | --- | --- |
| Addition or subtraction | Y = a + b – c | $S_{y}=\sqrt{{s_{a}}^{2}+{s_{b}}^{2}+{s_{c}}^{2}}$ |
| Multiplication or division | Y = (a x b) / c | $\frac{s_{y}}{y}=\sqrt{\left( \frac{S_{a}}{a} \right)^{2}+\left( \frac{S_{b}}{b} \right)^{2}+\left( \frac{s_{c}}{c} \right)^{2}}$ |

Hence the uncertainty of the eta correction is propagated as follows:

$S_{\left( \frac{\delta_{M}-\left( \delta_{S}-\eta\right)}{(1-\eta)} \right)}=S_{\delta_{eta}}=\sqrt{\left( \frac{S_{\eta}}{1-\eta} \right)^{2}+\left( \frac{\sqrt{{S_{\delta_{M}}}^{2}+\left( \left( \delta_{S}-\eta\right)*\sqrt{\left( \frac{S_{\delta_{S}}}{\delta_{S}} \right)^{2}+\left( \frac{S_{\eta}}{\eta} \right)^{2}} \right)^{2}}}{\delta_{M}-\left( \delta_{S}-\eta\right)} \right)^{2}}*\frac{\delta_{M}-\left( \delta_{S}-\eta\right)}{(1-\eta)}$ Eq. 2

Where S_δeta_, S_δM_, S_δS_ and S_η_ are the standard deviations of the eta corrected value, the measured value, the average value of the drift corrected working standard and the eta value, respectively.

In the next step the values are scaled to the VPDB scale via the working standard “Cecily2019a” with *δ*^13^C_VPDB_ and *δ*^18^O_VPDB_ values of -4.045 ± 0.005 and ‰ and -13.745 ± 0.008 ‰, respectively (Table 4), using the 1-point-calibration equation published by Paul et al. (2007):

$\delta_{T}=\left[ \frac{\left( \delta_{M}+1000 \right)*\left( \delta_{S(T)}+1000 \right)}{\left( \delta_{S(M)}+1000 \right)} \right]-1000$ Eq. 3

*δ*_T_, *δ*_M_, *δ*_S(T)_ and *δ*_S(M)_ are the delta values of the VPDB scaled sample, the measured sample, the value of the standard on the VPDB scale and the measured value of the standard, respectively.

Table 4: *δ*^13^C and *δ*^18^O values scaled to the VPDB scale using a 1-point calibration and associated propagated standard deviations.

| Sample/Standard Name | δ^13^C (VPDB) | δ^13^C (S.D.) | δ^18^O (VPDB) | δ^18^O (S.D.) |
| --- | --- | --- | --- | --- |
| Working Standard CECILY2019a | -4.0492 | 0.0164 | -13.7500 | 0.0247 |
| CECILY2019a | -4.0431 | 0.0168 | -13.7376 | 0.0238 |
| CECILY2019a | -4.0431 | 0.0161 | -13.7468 | 0.0254 |
| NBS-19 20220298 Batch 791 | 1.9526 | 0.0128 | -2.1933 | 0.0232 |
| NBS-19 20220298 Batch 791 | 1.9495 | 0.0131 | -2.1957 | 0.0263 |
| NBS-19 20220298 Batch 791 | 1.9423 | 0.0192 | -2.1986 | 0.0215 |
| RM8562 20220299 CO2 NIST837*100126 | -3.7442 | 0.0109 | -18.6804 | 0.0268 |
| RM8562 20220299 CO2 NIST837*100126 | -3.7537 | 0.0198 | -18.6907 | 0.0257 |
| RM8562 20220299 CO2 NIST837*100126 | -3.7413 | 0.0149 | -18.6848 | 0.0211 |
| RM8564 20220297 CO2 NIST837*002093 | -10.4684 | 0.0143 | -10.0852 | 0.0218 |
| RM8564 20220297 CO2 NIST837*002093 | -10.4803 | 0.0157 | -10.0866 | 0.0232 |
| RM8564 20220297 CO2 NIST837*002093 | -10.4688 | 0.0165 | -10.0786 | 0.0224 |
| Simon aliquot 20202178 | -37.9995 | 0.0159 | -35.8175 | 0.0364 |
| Simon aliquot 20202178 | -38.0128 | 0.0228 | -35.8028 | 0.0326 |
| Simon aliquot 20202178 | -38.0056 | 0.0123 | -35.8127 | 0.0355 |
| Working Standard CECILY2019a | -4.0493 | 0.0148 | -13.7473 | 0.0304 |
| CECILY2019a | -4.0399 | 0.0154 | -13.7365 | 0.0244 |
| CECILY2019a | -4.0463 | 0.0139 | -13.7506 | 0.0311 |

The uncertainties associated with the scaling equation are propagated using the following equation:

$S_{\left( \frac{\left( \delta_{M}+1000 \right)*\left( \delta_{S(T)}+1000 \right)}{\left( \delta_{S(M)}+1000 \right)} \right)}=S_{\delta T}=\left( \frac{\left( \delta_{M}+1000 \right)*\left( \delta_{S(T)}+1000 \right)}{\left( \delta_{S(M)}+1000 \right)} \right)*\sqrt{\left( \frac{S_{\delta S(M)}}{\left( \delta_{S(M)}+1000 \right)} \right)^{2}+\left( \frac{\left( \sqrt{\left( \frac{S_{\delta M}}{\left( \delta_{M}+1000 \right)} \right)^{2}+\left( \frac{S_{\delta S(T)}}{\left( \delta_{S(T)}+1000 \right)} \right)^{2}} \right)*\left( \delta_{M}+1000 \right)*\left( \delta_{S(T)}+1000 \right)}{\left( \delta_{M}+1000 \right)*\left( \delta_{S(T)}+1000 \right)} \right)^{2}}$ Eq. 4

Finally, an average delta value and an average propagated uncertainty of the three measurements are reported for each bellow filling (Table 5).

Table 5: fully corrected and scaled *δ*^13^C_VPDB_ and *δ*^18^O_VPDB_-CO_2_ values.

| Sample Name | δ^13^C (VPDB) | δ^13^C propagated uncertainty | δ^18^O (VPDB) | δ^18^O propagated uncertainty |
| --- | --- | --- | --- | --- |
| NBS19 | 1.95 | 0.015 | -2.20 | 0.024 |
| RM8562 | -3.75 | 0.015 | -18.69 | 0.025 |
| RM8564 | -10.47 | 0.016 | -10.08 | 0.022 |
| Simon QC | -38.01 | 0.017 | -35.81 | 0.035 |

**Value assignment of Working Standard “Cecily2019a”**

The working standard “Cecily2019a” used in this study is a pure CO_2_ gas aliquot that has been filled into a 5 L glass flask at a pressure of 1600 mbar. Throughout February 2022 ten NBS19 CO_2_ syntheses were done using ARAMIS, and these NBS19-CO2 gases were analyzed against the working standard in ten separate measurement sequences. Subsequently the working standard was scaled to the VPBD scale by setting the NBS19-CO_2_ δ^13^C and δ^18^O values to + 1.95 ‰ and – 2.20 ‰, respectively. Supplementary information 2 shows the unscaled NBS19 and Cecily2019a *δ*^13^C-CO_2_ and *δ*^18^O-CO_2_ values for each synthesis (and bellow filling) along with the propagated standard deviations that include the offline corrections described above, except the scaling correction. The average values and associated uncertainties are calculated using equations 5 and 6:

${NBS19}_{Average}=\frac{NBS19a+NBS19b+NBS19c\ldots+NBS19n}{n}$ Eq. 5

Where NBS19_average_ represents the average value of all NBS19 CO_2_ syntheses/bellow fillings (or Cecily2019a bellow fillings), individual values are given as NBS19a, NBS19b, NBS19c…etc., and n denotes the total number of CO_2_ syntheses/Bellow fillings.

$S_{NBS19(average)}={NBS19}_{average}*\sqrt{\left( \frac{\sqrt{\left( S_{NBS19a} \right)^{2}+\left( S_{NBS19b} \right)^{2}+\left( S_{NBS19c} \right)^{2}\ldots+\left( S_{NBS19n} \right)^{2}}}{Sum(NBS19a+NBS19b+NBS19c\ldots+NBS19n} \right)^{2}}$ Eq. 6

Where S_NBS19(average)_ is the average standard deviation of the average, and S_NBS19a, b, c, n_ are the propagated standard deviations of individually analysed CO_2_ syntheses/bellows fillings. Equation 5 gives identical average uncertainty values to those given by the NIST uncertainty machine (Lafarge and Possolo, 2015). Finally, Cecily2019a is scaled to the VPDB scale using equation 3 and the uncertainties are calculated using equation 4. The VPDB scaled *δ*^13^C-CO_2_ and *δ*^18^O-CO_2_ values of Cecily2109a are -4.045 ± 0.005 ‰ and -13.745 ± 0.005 0.007 ‰, respectively.

**Literature**

JCGM, 2008. Evaluation of measurement data - Guide to the expression of uncertainty in measurement. JCGM 100.

Lafarge, T., Possolo, A., 2015. The NIST Uncertainty Machine. NCSLI Measure 10, 20-27.

Paul, D., Grzegorz, S., István, F., 2007. Normalization of measured stable isotopic compositions to isotope reference scales – a review. Rapid Communications in Mass Spectrometry 21, 3006-3014.

Verkouteren, R.M., Assonov, S., Klinedinst, D.B., Brand, W.A., 2003. Isotopic metrology of carbon dioxide. II. Effects of ion source materials, conductance, emission, and accelerating voltage on dual-inlet cross contamination. Rapid Communications in Mass Spectrometry 17, 777-782.
